# Supplementary material for: Association between obstructive sleep apnoea and cancer: a cross-sectional, population-based study of the DISCOVERY cohort
Source: BMJ Open. 2023 Mar 3;13(3):e064501. doi: 10.1136/bmjopen-2022-064501 (PMC9990651; doi:10.1136/bmjopen-2022-064501)
Supplement: Author's manuscript [file bmjopen-2022-064501.draft_revisions.pdf]

BMJ Open is committed to open peer review. As part of this commitment we make the peer review history of every article we publish publicly available.

When an article is published we post the peer reviewers' comments and the authors' responses online. We also post the versions of the paper that were used during peer review. These are the versions that the peer review comments apply to.

The versions of the paper that follow are the versions that were submitted during the peer review process. They are not the versions of record or the final published versions. They should not be cited or distributed as the published version of this manuscript.

BMJ Open is an open access journal and the full, final, typeset and author-corrected version of record of the manuscript is available on our site with no access controls, subscription charges or pay-per-view fees (http://bmjopen.bmj.com).

If you have any questions on BMJ Open's open peer review process please email info.bmjopen@bmj.com

Obstructive sleep apnoea is associated with cancer– the population-based DISCOVERY study

Journal:	*BMJ Open*
Manuscript ID	bmjopen-2022-064501
Article Type:	Original research
Date Submitted by the Author:	09-May-2022
Complete List of Authors:	Palm, Andreas; Uppsala University, Department of Medical Sciences, Lung,- Allergy-, and Sleep Research; Region of Gävleborg Gävle Hospital, Centre for Research and Development<br>Theorell-Haglöw, J; Uppsala University Department of Medical Sciences, Lung,- Allergy-, and Sleep Research<br>Isaksson, Johan ; Uppsala University, Department of Immunology, Genetics and Pathology; Region of Gävleborg Gävle Hospital, Centre for Research and Development<br>Ljunggren, Mirjam; Uppsala University Department of Medical Sciences, Lung,- Allergy-, and Sleep Research<br>Sundh, Josefin; Örebro University Faculty of Medicine and Health<br>Ekström, Magnus; Lunds Universitet<br>Grote, Ludger; Goteborgs Universitet, Internal Medicine and Clinical Nutrition
Keywords:	SLEEP MEDICINE, ONCOLOGY, Adult thoracic medicine < THORACIC MEDICINE

SCHOLARONE™
Manuscripts

I, the Submitting Author has the right to grant and does grant on behalf of all authors of the Work (as defined in the below author licence), an exclusive licence and/or a non-exclusive licence for contributions from authors who are: i) UK Crown employees; ii) where BMJ has agreed a CC-BY licence shall apply, and/or iii) in accordance with the terms applicable for US Federal Government officers or employees acting as part of their official duties; on a worldwide, perpetual, irrevocable, royalty-free basis to BMJ Publishing Group Ltd ("BMJ") its licensees and where the relevant Journal is co-owned by BMJ to the co-owners of the Journal, to publish the Work in this journal and any other BMJ products and to exploit all rights, as set out in our licence.

The Submitting Author accepts and understands that any supply made under these terms is made by BMJ to the Submitting Author unless you are acting as an employee on behalf of your employer or a postgraduate student of an affiliated institution which is paying any applicable article publishing charge ("APC") for Open Access articles. Where the Submitting Author wishes to make the Work available on an Open Access basis (and intends to pay the relevant APC), the terms of reuse of such Open Access shall be governed by a Creative Commons licence – details of these licences and which Creative Commons licence will apply to this Work are set out in our licence referred to above.

Other than as permitted in any relevant BMJ Author's Self Archiving Policies, I confirm this Work has not been accepted for publication elsewhere, is not being considered for publication elsewhere and does not duplicate material already published. I confirm all authors consent to publication of this Work and authorise the granting of this licence.

For peer review only

Obstructive sleep apnoea is associated with cancer– the population-based DISCOVERY study

Andreas Palm, MD PhD[1,2], Jenny Theorell-Haglöw PhD[1], Johan Isakson MD[2], Mirjam Ljunggren MD PhD[1], Josefin Sundh MD PhD[3], Magnus Ekström MD PhD[4], Ludger Grote MD PhD[5,6]

[1]Department of Medical Sciences, Respiratory, Allergy and Sleep Research, Uppsala University, Box 256, SE-751 05, Uppsala, Sweden

[2]Centre for Research and Development, Uppsala University, Region of Gävleborg, Gävle Hospital, SE-80188, Gävle, Sweden

[3]Department of Respiratory Medicine, Faculty of Medicine and Health, Örebro University, Örebro, Sweden

[4]Department of Clinical Sciences, Respiratory Medicine and Allergology, Faculty of Medicine, Lund University, 221 84 Lund, Sweden

[5]Centre for Sleep and Wake Disorders, Sahlgrenska Academy, Gothenburg University, 413 90 Gothenburg, Sweden

[6]Sleep Disorders Center, Department of Pulmonary Medicine, Sahlgrenska University Hospital, Gothenburg, Sweden

Corresponding author: Andreas Palm, andreas.palm@medsci.uu.se

Running head: OSA and cancer

For peer review only

Keywords: OSA, cancer, hypoxia, register

Number of tables: 2; **Number of figures:** 2; **Number of references: 40**

Word count for body of abstract: 250**, text:** 2,934

For peer review only

Abstract

Objectives Nocturnal hypoxia in obstructive sleep apnoea (OSA) is a potential risk factor for cancer. We aimed to investigate the association between OSA measures and cancer prevalence in a large national patient cohort.

Design Cross-sectional study.

Settings 44 sleep centres in Sweden.

Participants 62,811 patients from the Swedish registry for positive airway pressure (PAP) treatment in OSA, linked to the national cancer registry and national socio-economic data (the DISCOVERY cohort).

Primary and Secondary Outcome Measures After propensity score matching for relevant confounders (anthropometric data, comorbidities, socio-economic status, smoking prevalence), sleep apnoea severity, measured as apnoea-hypopnoea index (AHI) or oxygen desaturation index (ODI), were compared between those with and without cancer diagnosis up to 5 years prior to PAP initiation. Subgroup analysis for cancer subtype was performed.

Results OSA patients with cancer (n=2,093) (29.8% females, age 65.3 (SD 10.1) years, BMI 30 (interquartile range 27-34) kg/m^2) had higher median AHI (n/h) (32(IQR 20-50) versus 30 (IQR 19-45), n/h, p=0.002) and median ODI (n/h) (28 (IQR 17-46) versus 26 (IQR 16-41), p<0.001) when compared with matched OSA patients without cancer. In subgroup analysis ODI was significantly higher in OSA patients with lung cancer (N=57; 38 (21-61) versus 27 (16-43), p=0.012)), prostate cancer (N=617; 28 (17-46) versus 24 (16-39), p=0.005), and malignant melanoma (N=170; 32 (17-46) versus 25 (14-41), p=0.015).

For peer review only

Conclusions OSA mediated intermittent hypoxia was independently associated with cancer prevalence in this large, national cohort. Future longitudinal studies are warranted to study the potential protective influence of OSA treatment on cancer incidence.

Strengths and limitations of the study

The size of the cohort with a large number of patients with verified cancer diagnosis and concomitant OSA.

High external validity since the Swedevox registry reflects almost 90% of new CPAP treatment starts in Sweden initiated by 44 sleep centers across the country and the cancer registry has almost 100 % coverage.

High internal validity since the Swedevox registry recently been validated against the medical record data entries and showed more than 95% correctness for age, BMI and AHI values.

Important lifestyle risk factors for cancer including smoking, physical activity or food preferences have not been captured on an individual level in our study but we included data on smoking prevalence in the different regions of Sweden to capture the potential influence of smoking on cancer prevalence.

Due to the cross-sectional design, our study cannot speculate about the causality between OSA and cancer.

For peer review only

Introduction

Patients with obstructive sleep apnoea (OSA) have recurring airway collapses during sleep with corresponding episodes of hypoxia. Animal models suggest an association between intermittent hypoxia and increased tumor growth, increased angiogenesis, changes in immune function, and increases in inflammation and oxidative stress (1). Several studies investigated the potential causal relationship between OSA and cancer (2-10) and some but not all report an increased prevalence of cancer in the OSA population studied. A recent meta-analysis provided evidence for a 40-50% increased risk ratio for cancer incidence in OSA patients (11).

As OSA is associated with several other risk factors for cancer disease, such as obesity, cardiometabolic disease, and lifestyle factors, the independent influence of OSA related hypoxia for an increased cancer risk remained unsolved. The validity of existing data may be in part hampered by the relatively low number of reported cancer cases, a potential reporting bias due to patient reported cancer diagnosis without the validation of independent sources, as well as by a selection bias due to single center studies.

In Sweden, 85% of patients with OSA receiving CPAP are reported to the Swedevox registry (12, 13) and almost all patients diagnosed with cancer are reported to the mandatory governmental Cancer Registry (14). The Swedish system with personal identity numbers (PIN) (15) allows for cross-linkage of various registries, creating large data bases with reliable data quality. Thereby, our current study aimed to overcome some of the limitations mentioned above by exploring the association between measures of OSA and cancer prevalence in a large, multicentric national cohort of OSA patients referred for PAP-

For peer review only

treatment using validated data from the national cancer registry for verified cancer diagnosis and subclassification.

For peer review only

MATERIAL AND METHODS

Study design and population

This was a national, population-based, cross-sectional study from the "course of DIsease in patients reported to Swedish CPAP, Oxygen and VEntilator RegistrY" (DISCOVERY) cohort. The study has been detailed elsewhere (13). In this study, data on patients aged ≥ 16 years with OSA on CPAP therapy, correctly and prospectively reported to the Swedevox registry (12) between 1st of July 2010 and 31th December 2017 were cross-linked with data from the mandatory National cancer registry (16) for information about cancer diagnosis, the Prescribed Drug Registry with information about all prescriptions of medications (17) and the National Patient Registry (NPR) covering data and diagnosis from all inpatient and outpatient visits at governmental hospitals (18). Socio-economic data (marital status, education level) were covered from Statistics Sweden (19). An overview of the study flow is presented in Figure 1.

End-point variables and co-variables

The primary variable of interest was captured by the diagnose codes "all-cause cancer" (ICD-10 code C00-99) up to five years prior initiation of CPAP therapy. Additional information was captured from cause-specific cancer: Ear-, neck-, and throat cancer (ENT) (C1-14), colon (C18), lung (C34), malignant melanomas (C43), breast (C50), prostate (C61), and urinary (C66-67). Information on "Skin cancer" (C44) other than malign melanoma was excluded. Information on sex, body mass index (BMI), apnoea-hypopnoea index (AHI), oxygen desaturation index (ODI), Epworth sleepiness scale score (ESS), and date of initiation of treatment were derived from the Swedevox registry (12, 13). Comorbid heart failure (ICD-10

codes I11, I42 and I50) and ischemic heart disease (ICD-10 code I20-25) was derived from NPR (18). Comorbid obstructive lung disease (OLD) was classified according to accepted practice by ≥ 2 collections of anti-obstructive drugs (ATC code R03) ≤12 months prior to initiation of CPAP and comorbid diabetes was classified by ≥1 collection of antidiabetic drugs (A10) ≤6 months prior initiation of CPAP therapy was registered in the national Prescribed Drug Registry (17). Information on educational level was obtained from Statistic Sweden and was categorized as low (<=9 years), medium (10-12 years), or high (≥13 years), corresponding to compulsory school, secondary school, and post-secondary school (college and university), respectively. The Swedevox registry does not contain information about individual smoking data. A marker for smoking exposure was generated from information on annual smoking prevalence between 2006 and 2018, in each county according to Statistics Sweden's Living Conditions Surveys (ULF/ SILC) (20). Counties were trichotomized according to mean smoking rate (8.3-12.0%, 12.1-13.5%, and 13.6-14.8%).

Statistical analyses

OSA severity was graded by the AHI and ODI both as continuous and categorical variables (AHI <15 (mild OSA), 15-<30 (moderate OSA) and ≥30 (severe OSA)). Clinical cut-off values for ODI severity are not defined and we therefore classified ODI into quartiles when used as categorical variable. Association between sleep recording variables and all-cause cancer diagnosis ≤5 years prior to the initiation of CPAP therapy was analyzed. In detail, the relationship between prevalent all-cause cancer and cause-specific cancer and AHI and ODI were analyzed using propensity score matched models with a case:control ratio 1:1. For propensity score matching, independent co-variables were used as anthropometrics (age, sex, BMI), presence of comorbidities at baseline (ischemic heart disease, heart failure,

For peer review only

diabetes, OLD), socio-economic data (civil status and educational level), start year of CPAP treatment and smoking prevalence (by county, trichotomized). These factors were chosen based on direct acyclic graphs using the browser-based environment DAGitty (www. dagitty.net) [21]. In addition, we performed a sensitivity analysis applying logistic regression analysis in the entire baseline population to predict "all cause cancer diagnosis" independent of all mentioned covariates used for the propensity matching procedure.

Normally distributed continuous data were expressed as mean ± SD and skewed distributed data were expressed as median with interquartile range (IQR). Categorical data were presented as frequencies and percentages. Differences between groups was analyzed using chi-2 test for categorical variables and Student´s t-test for continuous variables. A p-value <0.05 was considered statistically significant. Statistical analyses were conducted using the software packages Stata, version 17.0 (StataCorp LP; College Station, TX 77845 USA).

Patient and public involvement

Neither patients nor public were involved in the design, conduct, or reporting of the research.

For peer review only

Results

Patient characteristics

Clinical data, in particular age, and frequency of comorbidities varied significantly between unmatched cases (N=2,933) and controls (N=59,878). The final propensity score-matched study population consisted of 2,096 patients with cancer (29.8% females, age 65.3 (SD 10.1) years, BMI 30 (IQR 27-34) kg/m^2) and 2,096 matched OSA patients without cancer (**Table 1**).

Table 1. Characteristics of unmatched and propensity score matched study population. Matched by anthropometric variables (sex, age, BMI), comorbidities at baseline (ischemic heart disease, heart failure, diabetes mellitus and obstructive lung disease), socioeconomic factors (civil status and educational level), start year of CPAP treatment and county trichotomized by smoking prevalence.

	Unmatched			Matched		
	No cancer N=59,878	All-cause cancer N=2,933	p-value	No cancer N=2,093	All-cause cancer N=2,093	p-value
Sex, females (%)	17,647 (29.5%)	903 (30.8%)	0.13	661 (31.6%)	623 (29.8%)	0.20
Age, years	56.8 (12.5)	65.5 (10.2)	<0.001	65.3 (10.2)	65.3 (10.1)	0.91
BMI, kg/m2	31 (28-35)	30 (27-34)	<0.001	30 (27-34)	30 (27-34)	0.71
ESS, units	10.3 (5.0)	9.9 (4.9)	<0.001	9.6 (4.9)	10.0 (4.9)	0.02
Ischemic heart disease	5,671 (9.5%)	425 (14.5%)	<0.001	311 (14.9%)	309 (14.8%)	0.93
Heart failure	3,263 (5.4%)	277 (9.4%)	<0.001	180 (8.6%)	186 (8.9%)	0.74
Diabetes mellitus	8,548 (14.3%)	526 (17.9%)	<0.001	360 (17.2%)	366 (17.5%)	0.81
OLD	7,610 (12.7%)	462 (15.8%)	<0.001	325 (15.5%)	327 (15.6%)	0.93
Civil status			<0.001			0.46
Married	31,926 (53.5%)	1,771 (61.0%)		1,300 (62.1%)	1,280 (61.2%)	
Unmarried	14,675 (24.6%)	392 (13.5%)		289 (13.8%)	275 (13.1%)	
Divorced	10,561 (17.7%)	520 (17.9%)		340 (16.2%)	378 (18.1%)	
Widower/widow	2,529 (4.2%)	219 (7.5%)		164 (7.8%)	160 (7.6%)	
Level of education			<0.001			0.85
Low (≤9 years)	10,547 (21.7%)	605 (26.1%)		540 (25.8%)	546 (26.1%)	
Medium (10-12 years)	25,065 (51.5%)	1,061 (45.8%)		977 (46.7%)	959 (45.8%)	
High (≥13 years)	13,060 (26.8%)	653 (28.2%)		576 (27.5%)	588 (28.1%)	
AHI, events/hour	30 (19-48)	32 (20-49)	0.006	30 (19-45)	32 (20-50)	0.002
AHI category, events/hour			0.011			0.27
AHI <5	659 (1.1%)	20 (0.7%)		15 (0.7%)	14 (0.7%)	

For peer review only

AHI 5-14.9	8,181 (14.0%)	359 (12.6%)		272 (13.0%)	247 (11.8%)	
AHI 15-29.9	19,315 (33.0%)	929 (32.6%)		718 (34.3%)	682 (32.6%)	
AHI ≥30	30,332 (51.9%)	1,543 (54.1%)		1,088 (52.0%)	1,150 (54.9%)	
ODI, events/hour	27 (16-45)	28 (17-45)	0.084	26 (16-41)	28 (17-46)	<0.001
ODI, quartiles, events/hour			0.025			0.001
ODI 0-15.9	13,540 (22.9%)	595 (20.4%)		501 (23.9%)	433 (20.7%)	
ODI 16-27.9	14,862 (25.1%)	761 (26.2%)		587 (28.0%)	576 (27.5%)	
ODI 28-44.9	13,865 (23.4%)	704 (24.2%)		569 (27.2%)	546 (26.1%)	
ODI ≥45	16,975 (28.7%)	850 (29.2%)		436 (20.8%)	538 (25.7%)	

Data are presented as mean /SD) or median (IQR) dor continuos measures, and n (%) for categorical measures.
AHI: apnea-hypopnoea index; BMI: body mass index; ESS: Epworth sleepiness scale; ODI: oxygen desaturation index; OLD: obstructive lung disease

After matching procedure, clinical characteristics did not differ between cases and controls except for the Epworth Sleepiness Scale score which was slightly elevated in cases compared with controls (ESS score 10.0 (SD 4.9) versus 9.6 (SD 4.9), p=0.02, respectively).

Sleep apnoea severity in OSA patients with and without cancer

Cases with cancer disease had higher AHI (32 (20-50) versus 30 (19-45), n/h, p=0.002) and ODI (28 (17-46) versus 26 (16-41), p<0.001) when compared with matched controls without cancer (**Table 1** and **Figure 2**). Severe nocturnal intermittent hypoxia (ODI quartile IV (>45n/h)) was significantly more prevalent in cases (25.7%) compared to controls (20.8%), p=0.001 for ODI categories. Corresponding numbers for the highest AHI class (≥30 n/h) were 54.9% in cases and 52.4% in controls but the differences in AHI categories did not reach statistical significance.

Subgroup analysis confirmed that ODI was significantly higher in OSA patients with lung cancer (N=57, 38 (21-61) versus 27 (16-43), p=0.012)), prostate cancer (N=617, 28 (17-46) versus 24 (16-39), p=0.005), and malignant melanoma (N=170, 32 (17-46) versus 25 (14-41),

For peer review only

p=0.015) when compared with corresponding OSA patients without cancer (**Table 2 and 3**).

The remaining cancer subtypes did not differ in OSA severity measures.

For peer review only

Table 2. Frequency of cause specific cancer (lung cancer, ENT cancer and breast cancer) prevalence ≤5 years prior in relation to AHI and ODI in propensity score matched cohorts. AHI and ODI are expressed as both continuous and categorized variables.

	**No lung cancer** N=57	**Lung cancer** N=57	**p-value**	**No ENT cancer** N=31	**ENT cancer** N=31
AHI, events/hour	28 (18-44)	38 (25-56)	0.019	29 (18-46)	30 (17-41)
AHI category, events/hour			0.10		
AHI <5	0	0		0	0
AHI 5-14.9	8 (14.0%)	6 (10.5%)		1 (3.2%)	7 (22.6%)
AHI 15-29.9	21 (36.8%)	12 (21.1%)		15 (48.4%)	8 (25.8%)
AHI ≥30	28 (49.1%)	39 (68.4%)		15 (48.4%)	16 (51.6%)
ODI, events/hour	27 (16-43)	38 (21-61)	0.012	24 (15-46)	23 (13-39)
ODI, quartiles, events/hour			0.034		
ODI 0-15.9	13 (22.8%)	6 (10.5%)		8 (25.8%)	11 (35.5%)
ODI 16-27.9	17 (29.8%)	17 (29.8%)		10 (32.3%)	6 (19.4%)
ODI 28-44.9	15 (26.3%)	9 (15.8%)		5 (16.1%)	10 (32.3%)
ODI ≥45	12 (21.1%)	25 (43.9%)		8 (25.8%)	4 (12.9%)

	**No Breast cancer** N=162	**Breast cancer** N=162	**p-value**
	28 (17-46)	28 (18-46)	0.49
AHI, events/hour			0.59
AHI category, events/hour	4 (2.5%)	2 (1.2%)	
AHI <5	29 (17.9%)	24 (14.8%)	
AHI 5-14.9	51 (31.5%)	60 (37.0%)	
AHI 15-29.9	78 (48.1%)	76 (46.9%)	
AHI ≥30	24 (14-39)	27 (17-49)	0.26
ODI, events/hour			0.30
ODI, quartiles, events/hour	49 (30.2%)	38 (23.5%)	
ODI 0-15.9	39 (24.1%)	49 (30.2%)	
ODI 16-27.9	40 (24.7%)	34 (21.0%)	
ODI 28-44.9	34 (21.0%)	41 (25.3%)	
ODI ≥45			

Data are presented as mean (SD) or median (IQR) for continuous measures, and n (%) for categorical measures.
AHI: apnoea-hypopnoea index; ODI: oxygen desaturation index

For peer review only

Table 3. Frequency of cause specific cancer (prostate cancer, colon cancer, urinary tract cancer and malignant melanoma) prevalence ≤5 years prior in relation to AHI and ODI in propensity score matched cohorts. AHI and ODI are expressed as both continuous and categorized variables.

	**No prostate cancer** N=617	**Prostate cancer** N=617	**p-value**	**No colon cancer** N=123	**Colon cancer** N=123	**p-value**
AHI, events/hour	**28 (19-44)**	**33 (20-50)**	**0.006**	33 (21-53)	30 (21-48)	0.41
AHI category, events/hour			**0.035**			0.31
AHI <5	**4 (0.6%)**	**2 (0.3%)**		4 (3.3%)	2 (1.6%)	
AHI 5-14.9	**70 (11.3%)**	**66 (10.7%)**		10 (8.1%)	13 (10.6%)	
AHI 15-29.9	**251 (40.7%)**	**208 (33.7%)**		35 (28.5%)	46 (37.4%)	
AHI ≥30	**292 (47.3%)**	**341 (55.3%)**		74 (60.2%)	62 (50.4%)	
ODI, events/hour	**24 (16-39)**	**28 (17-46)**	**0.005**	28 (18-50)	26 (17-43)	0.47
ODI, quartiles, events/hour			**0.009**			0.71
ODI 0-15.9	**147 (23.8%)**	**129 (20.9%)**		24 (19.5%)	26 (21.1%)	
ODI 16-27.9	**210 (34.0%)**	**170 (27.6%)**		34 (27.6%)	36 (29.3%)	
ODI 28-44.9	**139 (22.5%)**	**161 (26.1%)**		28 (22.8%)	32 (26.0%)	
ODI ≥45	**121 (19.6%)**	**157 (25.4%)**		37 (30.1%)	29 (23.6%)	

For peer review only

	No urinary tract cancer N=93	Urinary tract cancer N=93	p-value	No malignant melanoma N=170	Malignant melanoma N=170	p-value
AHI, events/hour	31 (19-47)	35 (24-52)	0.15	28 (17-45)	35 (23-52)	0.003
AHI category, events/hour			0.40			0.072
AHI <5	1 (1.1%)	1 (1.1%)		2 (1.2%)	1 (0.6%)	
AHI 5-14.9	13 (14.0%)	6 (6.5%)		25 (14.7%)	15 (8.8%)	
AHI 15-29.9	26 (28.0%)	30 (32.3%)		65 (38.2%)	53 (31.2%)	
AHI ≥30	53 (57.0%)	56 (60.2%)		78 (45.9%)	101 (59.4%)	
ODI, events/hour	28 (16-47)	32 (21-50)	0.11	25 (14-41)	32 (17-46)	0.015
ODI, quartiles, events/hour			0.67			0.13
ODI 0-15.9	20 (21.5%)	14 (15.1%)		48 (28.2%)	32 (18.8%)	
ODI 16-27.9	26 (28.0%)	25 (26.9%)		47 (27.6%)	45 (26.5%)	
ODI 28-44.9	22 (23.7%)	25 (26.9%)		44 (25.9%)	50 (29.4%)	
ODI ≥45	25 (26.9%)	29 (31.2%)		31 (18.2%)	43 (25.3%)	

Data are presented as mean (SD) or median (IQR) for continuous measures, and n (%) for categorical measures.
AHI: apnoea-hypopnoea index; ENT: Ear-, nose-, and throat

For peer review only

In sensitivity analysis including the entire cohort (N=62,811, logistic regression analysis with cancer as the independent variable) we identified significant odds ratios for ODI and AHI (1.05 (1.03-1.07) and 1.04 (1.02-1.07) per 10 units, p<0.001, respectively) confirming the data obtained by propensity score matching.

Discussion

Main findings

Our study identified several important findings. First, OSA is associated with cancer prevalence independent of a substantial number of important confounders. The added risk is small and predominantly seen in patients with severe intermittent hypoxia during sleep measured by ODI rather than in those with frequent respiratory events during sleep measured by AHI. Second, the association between intermittent hypoxia and cancer was identified in lung cancer, malignant melanoma, and prostate cancer. Our study underlines the importance of further studies to investigate if preventing and treating sleep apnoea may have beneficial effects on cancer incidence and any outcomes of cancer treatment.

Cancer prevalence in other cohort studies

Results from previous studies on OSA and cancer are conflicting. One early Spanish multi-center study (4,910 patients) identified an increased cancer incidence in OSA patients with an overall odds ratio of 1.45 (95% CI 1.1-1.9) (2). In a Canadian cohort study (n= 10,149) from a single sleep clinic at an academic hospital, 520 patients had a cancer diagnosis at baseline

For peer review only - http://bmjopen.bmj.com/site/about/guidelines.xhtml

For peer review only

and 627 patients of the cancer-free patients at baseline were reported with incident cancer at follow-up (7.8 years) (10). No association between severity of OSA and cancer was found for prevalent or incident cancer. In a twice as large European cross-sectional study (n=19,556 of whom 388 self-reported cancer diagnosis at time of sleep study), an association between AHI and ODI was found in women but not in men (8). In a recent French cohort study (n=8,748, 5.8-year follow-up, 718 incident cancer cases), an association between nocturnal hypoxia, defined as the percent of night spent with saturation <90%, and incident cancer was found while there were no associations between AHI or ODI and incident cancer after adjustment for confounders (9). A recent meta-analysis of 12 studies including 184,915 patients with OSA (n=75,367 and 3,805 cancer cases) and patients without OSA (n=109,548 and 2,110 cancer cases) identified a dose dependent increased risk of incident all-cause cancer with an odds ratio of 1.14 (95% CI: 1.04-1.25, p=0.006) for mild OSA, 1.36 (1.32-1.92;p <0.001) for moderate OSA and 1.59 (95% CI: 1.45-1.74; P < 0.001) for severe OSA (11). Unfortunately, this meta-analysis did not evaluate the association between the amount of OSA related hypoxic measures and cancer incidence. In this context, our cross-sectional data in one of the largest OSA patient population so far is in line with previous findings. OSA appear to be associated with cancer, but effect sizes are generally small and large cohorts are needed to detect any independent effect of OSA on cancer incidence. It cannot be excluded that other confounders, not yet taken into consideration in any of the published analyses, may further explain part of the effect sizes accounted for OSA. Notably, several studies show that OSA measures reflecting hypoxic burden appear to better predict the risk for cancer when compared with AHI as traditional OSA severity marker (8, 9). Due to the exact classification of cancer type through the Swedish national registry we were able to perform subtype analysis on the influence of OSA related cancers. We identified more

For peer review only - http://bmjopen.bmj.com/site/about/guidelines.xhtml

severe OSA in lung cancer, in malignant melanoma and in prostate cancer which is in line with previous study results (22-25). In contrast, we did not identify increased prevalence of OSA in patients with breast cancer (26, 27) .

Potential mechanisms between the association of OSA and cancer

Previous studies have explored potential mechanistic pathways for the association between OSA and cancer. Experimental studies mimicking OSA by using an intermittent hypoxia model, show that intermittent hypoxia promotes incident tumor cell transition, tumor growth, and metastasis. Several in vitro cell models identified intermittent hypoxia to promote tumor cell growth in different cell lines (28). Hypoxia inducible factor (HIF) may activate vascular endothelial growth factor (VEGF) and promote vascular angiogenesis important for tumor growth (29). In vivo mouse models showed that melanoma metastasis was increased in animals exposed to intermittent hypoxia mimicking severe OSA (30). Although these data suggest a possible OSA-related mechanistic pathway for cancer growth and aggressiveness, further studies are needed to explore if and how OSA promote initial occurrence of cancer. In this context, obesity is an important confounder for the association between OSA and cancer. Obesity in the general population has nearly doubled in the last 20 years and by 2017, 17% of the Swedish adult population are obese defined as BMI exceeding 30 kg/m^2 (31). Presence of OSA is closely linked to obesity (32) and in Sweden, the mean BMI when initiating continuous positive airway pressure (CPAP) therapy is 32 kg/m^2 (12). Several cancer forms, i.e. breast-, colon-, kidney-, pancreas-, and esophageal cancer, are also associated with higher BMI (33). As both age standardized cancer incidence (34) as well as

For peer review only - http://bmjopen.bmj.com/site/about/guidelines.xhtml

prevalence of obesity are increasing (31) in parallel, we carefully controlled for both factors in our analysis.

Inflammation may be considered as a third potential pathway for the link between OSA and cancer. OSA is shown to increase systemic inflammation with increased levels of hsCRP, IL-6 and TNF-α (35, 36). Systemic inflammation has been linked to risk of cancer/cancer development but also tumor growth (37). In addition, low degree of inflammation has been identified in adipocytes which may contribute to the overall increased inflammation in obesity. Further studies linking markers of inflammation to cancer incidence in OSA populations are of particular interest.

Our study has several strengths. First, the study has a high validity as we included a large number of patients with verified cancer diagnosis and concomitant OSA. The National cancer registry has the highest quality due to regularly performed validation studies by the National Social Welfare Board. The sleep apnoea related data within the Swedevox registry has recently been validated against the medical record data entries and showed more than 95% correctness for age, BMI and AHI values (38). Second, our study cohort is representative for studying the association between OSA and cancer prevalence. The Swedevox registry reflects almost 90% of new CPAP treatment starts in Sweden initiated by 44 sleep centers across the country (12). The national cancer registry has almost 100% coverage (16). Socio-economic data are based on information from the National Tax Agency. No patient was lost due to missing data in the national registries. Third, we used a strong analysis methodology with sufficient power due to the large number of cases. The propensity score matching of cases and controls adjusting for a large number of important confounders can be viewed as close to a randomized study setting.

For peer review only - http://bmjopen.bmj.com/site/about/guidelines.xhtml

Nonetheless study limitations need also to be addressed. First, sleep apnoea within the Swedevox registry is almost exclusively classified by polygraphy and not by polysomnography. However, device methodology is very homogenous in Sweden and national guidelines for the analysis of polygraphic recordings are published (39). Polygraphic recording may underestimate the actual AHI classification, but to a lesser extent the ODI count. This may in part explain why ODI is a stronger predictor of cancer prevalence than AHI. Second, we do not have other measures of nocturnal hypoxia, for example the time spent in saturation below 90% or hypoxic burden analysis of the saturation curve, recently described as a significant predictor of cancer in OSA patients (2, 8, 9). Third, important lifestyle risk factors for cancer including smoking, physical activity or food preferences have not been captured on an individual level in our study. However, we have a strong classification of socio-economic factors. We also included data on smoking prevalence in the different regions of Sweden to capture the potential influence of smoking on cancer prevalence. Research into the impact of socioeconomic factors on cancer incidence typically find a larger prevalence of solid tumors in groups with less fortunate social standing with breast cancer being the sole outlier. The single most important variable in cancer incidence is tobacco smoking where large differences are seen between socioeconomic groups (40). In case of lacking information on individual patient data for such risk factors the use of socioeconomic stratification represents the best available method to adjust for this. Fourth, our study included only PAP treated OSA patients reflecting the upper range of OSA severity and not the entire OSA spectrum. This patient selection may have limited our possibility to fully assess the dose response relationship between OSA severity and cancer prevalence. However, the OSA patient group with severe intermittent hypoxia demonstrated the

For peer review only - http://bmjopen.bmj.com/site/about/guidelines.xhtml

strongest risk increase for cancer prevalence. Finally, due to the cross-sectional study design, our study cannot speculate about the causality between OSA and cancer.

Clinical implications and research agenda

Our study provides further cross-sectional evidence to the important clinical question on the association between OSA and cancer in OSA patients. Even if we did not analyze longitudinal data on the incidence of cancer, we see a significant yet limited effect size for OSA on the risk for prevalent cancer. More importantly, future analysis needs to focus on the effect of OSA treatment with PAP on cancer incidence and cancer survival.

Conclusion

OSA mediated intermittent hypoxia is an independent risk factor for cancer in this large, national OSA patient cohort. Future longitudinal studies are warranted to study the potential influence of OSA treatment on cancer incidence in patients with OSA.

ACKNOWLEDGEMENTS

Funding: AP was supported by grants from Gävle Cancer Foundation (grant number N/A), the Swedish Society for Sleep Research and Sleep Medicine (grant number N/A), Centre for Research and Development, Uppsala University/Region Gävleborg (grant number N/A), Bror Hjerpstedt´s Foundation (grant number N/A) and Uppsala Heart and Lung Foundation (grant number N/A) and Regional Research Council in Mid Sweden (RFR-931234). JTH was supported by grant from Swedish Heart and Lung Foundation (20190607, 20190611). ME

For peer review only - http://bmjopen.bmj.com/site/about/guidelines.xhtml

was supported by unrestricted grants from the Swedish Research Council (Dnr 2019-02081). LG was supported by the Swedish Heart and Lung Foundation (20180567, 20210529).

Competing interests

No conflicts of interest exist for the authors regarding the submitted manuscript.

Data sharing statement

The steering committee of the Swedevox quality registry will consider reasonable requests for the sharing of deidentified patient level data. Requests should be made to the corresponding author.

Author contributions

AP, JTH, JI, MJ, JS, ME and LG contributed to the conception and design of the study. AP performed statistical analyses and all authors verified the underlying data. AP and LG wrote the first draft. All authors had full access to all the data in the study and had final responsibility for the decision to submit for publication. All authors participated in data interpretation, drafting of the manuscript, and final approval for submission.

Ethics Statement

The study protocol was approved by the Ethics Committee at the Medical Faculty at Lund University and the Swedish Ethical Review Authority, Dnr. 2018/51, 2019/01420 and 2021/04984. According to Swedish law, patients entering a national quality registry like Swedevox are informed, can withdraw from registration at any time, but do not sign an informed consent.

For peer review only

For peer review only - http://bmjopen.bmj.com/site/about/guidelines.xhtml

REFERENCES

1. Almendros I, Montserrat JM, Torres M, Bonsignore MR, Chimenti L, Navajas D, et al. Obesity and intermittent hypoxia increase tumor growth in a mouse model of sleep apnea. Sleep medicine. 2012;13(10):1254-60. Epub 2012/11/15. doi: 10.1016/j.sleep.2012.08.012. PubMed PMID: 23149216.
2. Campos-Rodriguez F, Martinez-Garcia MA, Martinez M, Duran-Cantolla J, Pena Mde L, Masdeu MJ, et al. Association between obstructive sleep apnea and cancer incidence in a large multicenter Spanish cohort. American journal of respiratory and critical care medicine. 2013;187(1):99-105. Epub 2012/11/17. doi: 10.1164/rccm.201209-1671OC. PubMed PMID: 23155146.
3. Christensen AS, Clark A, Salo P, Nymann P, Lange P, Prescott E, et al. Symptoms of sleep disordered breathing and risk of cancer: a prospective cohort study. Sleep. 2013;36(10):1429-35. Epub 2013/10/02. doi: 10.5665/sleep.3030. PubMed PMID: 24082302; PubMed Central PMCID: PMC3773192.
4. Nieto FJ, Peppard PE, Young T, Finn L, Hla KM, Farre R. Sleep-disordered breathing and cancer mortality: results from the Wisconsin Sleep Cohort Study. American journal of respiratory and critical care medicine. 2012;186(2):190-4. Epub 2012/05/23. doi: 10.1164/rccm.201201-0130OC. PubMed PMID: 22610391; PubMed Central PMCID: PMC3406081.
5. Chang WP, Liu ME, Chang WC, Yang AC, Ku YC, Pai JT, et al. Sleep apnea and the subsequent risk of breast cancer in women: a nationwide population-based cohort study. Sleep medicine. 2014;15(9):1016-20. Epub 2014/08/03. doi: 10.1016/j.sleep.2014.05.026. PubMed PMID: 25085620.
6. Chen JC, Hwang JH. Sleep apnea increased incidence of primary central nervous system cancers: a nationwide cohort study. Sleep medicine. 2014;15(7):749-54. Epub 2014/06/04. doi: 10.1016/j.sleep.2013.11.782. PubMed PMID: 24891080.
7. Gozal D, Farre R, Nieto FJ. Putative Links Between Sleep Apnea and Cancer: From Hypotheses to Evolving Evidence. Chest. 2015;148(5):1140-7. Epub 2015/05/29. doi: 10.1378/chest.15-0634. PubMed PMID: 26020135; PubMed Central PMCID: PMC4631033.
8. Pataka A, Bonsignore MR, Ryan S, Riha RL, Pepin JL, Schiza S, et al. Cancer prevalence is increased in females with sleep apnoea: data from the ESADA study. The European respiratory journal. 2019;53(6). Epub 2019/05/22. doi: 10.1183/13993003.00091-2019. PubMed PMID: 31109987.
9. Justeau G, Gervès-Pinquié C, Le Vaillant M, Trzepizur W, Meslier N, Goupil F, et al. Association Between Nocturnal Hypoxemia and Cancer Incidence in Patients Investigated for OSA: Data From a Large Multicenter French Cohort. Chest. 2020;158(6):2610-20. Epub 2020/07/07. doi: 10.1016/j.chest.2020.06.055. PubMed PMID: 32629036.
10. Kendzerska T, Leung RS, Hawker G, Tomlinson G, Gershon AS. Obstructive sleep apnea and the prevalence and incidence of cancer. CMAJ : Canadian Medical Association journal = journal de l'Association medicale canadienne. 2014;186(13):985-92. Epub 2014/08/07. doi: 10.1503/cmaj.140238. PubMed PMID: 25096668; PubMed Central PMCID: PMC4162778.
11. Cheng H, Li D. Investigation into the association between obstructive sleep apnea and incidence of all-type cancers: a systematic review and meta-analysis. Sleep medicine. 2021;88:274-81. Epub 20210604. doi: 10.1016/j.sleep.2021.05.031. PubMed PMID: 34219029.
12. The Swedevox registry. Annual report 2020. 2021 [14 Sep 2021]. Available from: https://www.ucr.uu.se/swedevox/rapporter/arsrapporter/arsrapporter/arsrapport-2020.
13. Palm A, Agren K, Grote L, Ljunggren M, Midgren B, Sundh J, et al. Course of DISease In patients reported to the Swedish CPAP Oxygen and VEntilator RegistrY (DISCOVERY) with population-

For peer review only - http://bmjopen.bmj.com/site/about/guidelines.xhtml

based controls. BMJ Open. 2020;10(11):e040396. Epub 2021/01/15. doi: 10.1136/bmjopen-2020-040396. PubMed PMID: 33444200; PubMed Central PMCID: PMC7678356.
14. Barlow L, Westergren K, Holmberg L, Talback M. The completeness of the Swedish Cancer Register: a sample survey for year 1998. Acta oncologica (Stockholm, Sweden). 2009;48(1):27-33. Epub 2008/09/04. doi: 10.1080/02841860802247664. PubMed PMID: 18767000.
15. Ludvigsson JF, Otterblad-Olausson P, Pettersson BU, Ekbom A. The Swedish personal identity number: possibilities and pitfalls in healthcare and medical research. European journal of epidemiology. 2009;24(11):659-67. Epub 2009/06/09. doi: 10.1007/s10654-009-9350-y. PubMed PMID: 19504049; PubMed Central PMCID: PMC2773709.
16. Pukkala E, Engholm G, Højsgaard Schmidt LK, Storm H, Khan S, Lambe M, et al. Nordic Cancer Registries - an overview of their procedures and data comparability. Acta oncologica (Stockholm, Sweden). 2018;57(4):440-55. Epub 2017/12/12. doi: 10.1080/0284186x.2017.1407039. PubMed PMID: 29226751.
17. Wettermark B, Hammar N, Fored CM, Leimanis A, Otterblad Olausson P, Bergman U, et al. The new Swedish Prescribed Drug Register--opportunities for pharmacoepidemiological research and experience from the first six months. Pharmacoepidemiology and drug safety. 2007;16(7):726-35. Epub 2006/08/10. doi: 10.1002/pds.1294. PubMed PMID: 16897791.
18. Ludvigsson JF, Andersson E, Ekbom A, Feychting M, Kim JL, Reuterwall C, et al. External review and validation of the Swedish national inpatient register. BMC public health. 2011;11:450. Epub 2011/06/11. doi: 10.1186/1471-2458-11-450. PubMed PMID: 21658213; PubMed Central PMCID: PMC3142234.
19. Ludvigsson JF, Svedberg P, Olen O, Bruze G, Neovius M. The longitudinal integrated database for health insurance and labour market studies (LISA) and its use in medical research. European journal of epidemiology. 2019;34(4):423-37. Epub 2019/04/01. doi: 10.1007/s10654-019-00511-8. PubMed PMID: 30929112; PubMed Central PMCID: PMC6451717.
20. Living Conditions Surveys (ULF/SILC) 2019 [updated October 14, 2021February 10, 2022]. Available from: http://www.scb.se/le0101-en.
21. Lederer DJ, Bell SC, Branson RD, Chalmers JD, Marshall R, Maslove DM, et al. Control of Confounding and Reporting of Results in Causal Inference Studies. Guidance for Authors from Editors of Respiratory, Sleep, and Critical Care Journals. Annals of the American Thoracic Society. 2019;16(1):22-8. Epub 2018/09/20. doi: 10.1513/AnnalsATS.201808-564PS. PubMed PMID: 30230362.
22. Cheong AJY, Tan BKJ, Teo YH, Tan NKW, Yap DWT, Sia CH, et al. Obstructive Sleep Apnea and Lung Cancer: A Systematic Review and Meta-Analysis. Annals of the American Thoracic Society. 2022;19(3):469-75. doi: 10.1513/AnnalsATS.202108-960OC. PubMed PMID: 34792438.
23. Martinez-Garcia MA, Campos-Rodriguez F, Nagore E, Martorell A, Rodriguez-Peralto JL, Riveiro-Falkenbach E, et al. Sleep-Disordered Breathing Is Independently Associated With Increased Aggressiveness of Cutaneous Melanoma: A Multicenter Observational Study in 443 Patients. Chest. 2018;154(6):1348-58. Epub 20180727. doi: 10.1016/j.chest.2018.07.015. PubMed PMID: 30059679.
24. Chung WS, Lin CL. Sleep disorders associated with risk of prostate cancer: a population-based cohort study. BMC Cancer. 2019;19(1):146. Epub 20190213. doi: 10.1186/s12885-019-5361-6. PubMed PMID: 30760242; PubMed Central PMCID: PMC6375129.
25. Lee EJ, Suh JD, Cho JH. The incidence of prostate cancer is increased in patients with obstructive sleep apnea: Results from the national insurance claim data 2007-2014. Medicine. 2021;100(6):e24659. doi: 10.1097/md.0000000000024659. PubMed PMID: 33578596.
26. Choi JH, Lee JY, Han KD, Lim YC, Cho JH. Association between obstructive sleep apnoea and breast cancer: The Korean National Health Insurance Service Data 2007-2014. Sci Rep. 2019;9(1):19044. Epub 20191213. doi: 10.1038/s41598-019-55551-7. PubMed PMID: 31836779; PubMed Central PMCID: PMC6910956.
27. Madut A, Fuchsova V, Man H, Askar S, Trivedi R, Elder E, et al. Increased prevalence of obstructive sleep apnea in women diagnosed with endometrial or breast cancer. PloS one.

For peer review only - http://bmjopen.bmj.com/site/about/guidelines.xhtml

2021;16(4):e0249099. Epub 20210407. doi: 10.1371/journal.pone.0249099. PubMed PMID: 33826649; PubMed Central PMCID: PMC8026058.
28. Bhaskara VK, Mohanam I, Rao JS, Mohanam S. Intermittent hypoxia regulates stem-like characteristics and differentiation of neuroblastoma cells. PloS one. 2012;7(2):e30905. Epub 20120217. doi: 10.1371/journal.pone.0030905. PubMed PMID: 22363512; PubMed Central PMCID: PMC3281893.
29. Dong G, Lin XH, Liu HH, Gao DM, Cui JF, Ren ZG, et al. Intermittent hypoxia alleviates increased VEGF and pro-angiogenic potential in liver cancer cells. Oncol Lett. 2019;18(2):1831-9. Epub 20190618. doi: 10.3892/ol.2019.10486. PubMed PMID: 31423251; PubMed Central PMCID: PMC6607353.
30. Li L, Ren F, Qi C, Xu L, Fang Y, Liang M, et al. Intermittent hypoxia promotes melanoma lung metastasis via oxidative stress and inflammation responses in a mouse model of obstructive sleep apnea. Respiratory research. 2018;19(1):28. Epub 20180212. doi: 10.1186/s12931-018-0727-x. PubMed PMID: 29433520; PubMed Central PMCID: PMC5809953.
31. Hemmingsson E, Ekblom Ö, Kallings LV, Andersson G, Wallin P, Söderling J, et al. Prevalence and time trends of overweight, obesity and severe obesity in 447,925 Swedish adults, 1995-2017. Scandinavian journal of public health. 2020:1403494820914802. Epub 2020/05/01. doi: 10.1177/1403494820914802. PubMed PMID: 32349623.
32. Tishler PV, Larkin EK, Schluchter MD, Redline S. Incidence of sleep-disordered breathing in an urban adult population: the relative importance of risk factors in the development of sleep-disordered breathing. JAMA : the journal of the American Medical Association. 2003;289(17):2230-7. Epub 2003/05/08. doi: 10.1001/jama.289.17.2230. PubMed PMID: 12734134.
33. Park J, Morley TS, Kim M, Clegg DJ, Scherer PE. Obesity and cancer--mechanisms underlying tumour progression and recurrence. Nat Rev Endocrinol. 2014;10(8):455-65. Epub 2014/06/18. doi: 10.1038/nrendo.2014.94. PubMed PMID: 24935119; PubMed Central PMCID: PMC4374431.
34. Statistics on Cancer Incidence 2020: Socialstyrelsen, The National Board of Health and Welfare; 2021 [updated 15 Dec 202116 Jan 2022]. Available from: https://www.socialstyrelsen.se/globalassets/sharepoint-dokument/artikelkatalog/statistik/2021-12-7701.pdf.
35. Kheirandish-Gozal L, Gozal D. Obstructive Sleep Apnea and Inflammation: Proof of Concept Based on Two Illustrative Cytokines. Int J Mol Sci. 2019;20(3). Epub 20190122. doi: 10.3390/ijms20030459. PubMed PMID: 30678164; PubMed Central PMCID: PMC6387387.
36. Unnikrishnan D, Jun J, Polotsky V. Inflammation in sleep apnea: an update. Rev Endocr Metab Disord. 2015;16(1):25-34. doi: 10.1007/s11154-014-9304-x. PubMed PMID: 25502450; PubMed Central PMCID: PMC4346503.
37. Multhoff G, Molls M, Radons J. Chronic inflammation in cancer development. Front Immunol. 2011;2:98. Epub 20120112. doi: 10.3389/fimmu.2011.00098. PubMed PMID: 22566887; PubMed Central PMCID: PMC3342348.
38. Ekstrom M, Albrecht D, Andersson S, Grote L, Karrsten Rundstrom B, Palm A, et al. Validation of the Swedevox registry of continuous positive airway pressure, long-term mechanical ventilator and long-term oxygen therapy. ERJ Open Res. 2021;7(1). Epub 2021/02/04. doi: 10.1183/23120541.00340-2020. PubMed PMID: 33532458; PubMed Central PMCID: PMC7836467 D.
39. Grote Lea. National guidlines for for treatment of sleep apnea in adults 2021 [8 march 2022]. Available from: https://sesar.registercentrum.se/riktlinjer/nationellt-vardprogram-foer-behandling-av-soemnapne-hos-vuxna/p/HkusG6OcK.
40. Axelsson Fisk S, Lindström M, Perez-Vicente R, Merlo J. Understanding the complexity of socioeconomic disparities in smoking prevalence in Sweden: a cross-sectional study applying intersectionality theory. BMJ Open. 2021;11(2):e042323. Epub 20210211. doi: 10.1136/bmjopen-2020-042323. PubMed PMID: 33574148; PubMed Central PMCID: PMC7880088.

For peer review only - http://bmjopen.bmj.com/site/about/guidelines.xhtml

FIGURES INCLUDING LEGENDS

Figure 1. Study Flow Chart

For peer review only

For peer review only - http://bmjopen.bmj.com/site/about/guidelines.xhtml

Figure 2. Distribution of OSA patients with (red bars) and without (blue bars) cancer diagnosis in the four ODI quartiles (x-axis). Propensity-score matched analysis in 2,093 cases and 2,093 controls, ODI distribution differ significantly between cases and controls, p<0.001.

For peer review only

For peer review only - http://bmjopen.bmj.com/site/about/guidelines.xhtml

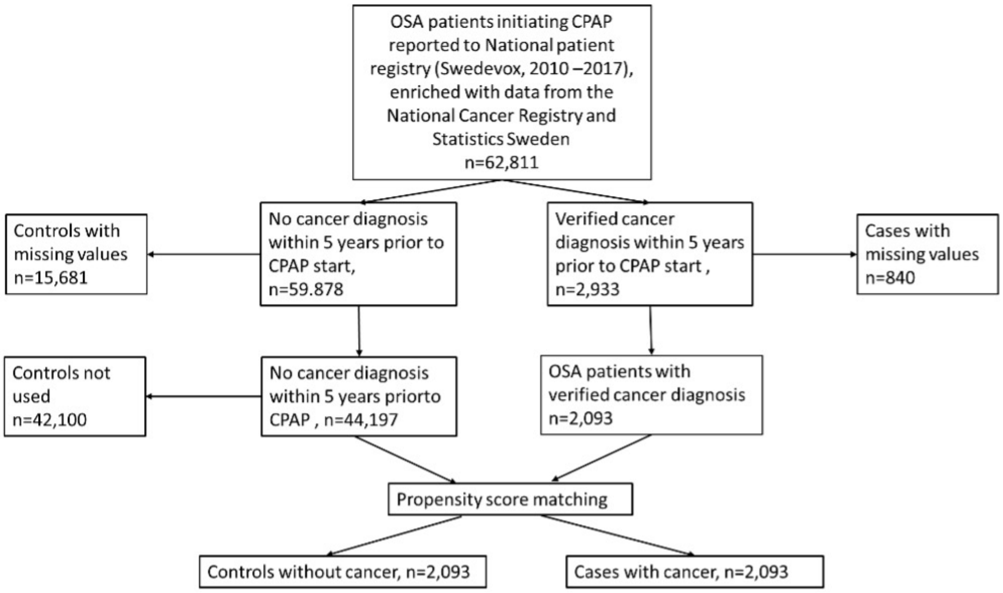

Figure 1. Study Flow Chart

167x99mm (150 x 150 DPI)

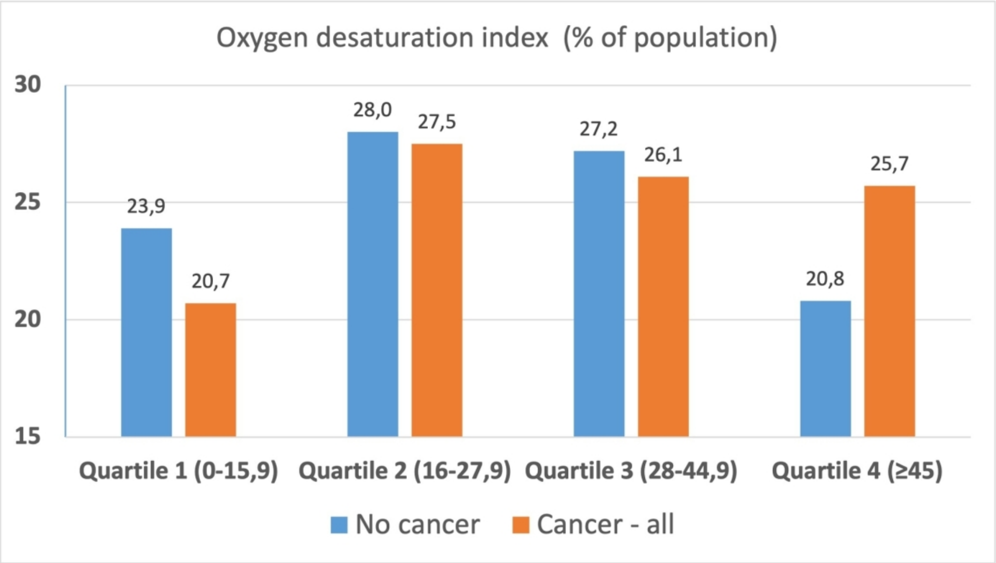

Figure 2. Distribution of OSA patients with (red bars) and without (blue bars) cancer diagnosis in the four ODI quartiles (x-axis). Propensity-score matched analysis in 2,093 cases and 2,093 controls, ODI distribution differ significantly between cases and controls, p<0.001.

160x91mm (300 x 300 DPI)

Reporting checklist for cross sectional study.

Based on the STROBE cross sectional guidelines.

Instructions to authors

Complete this checklist by entering the page numbers from your manuscript where readers will find each of the items listed below.

Your article may not currently address all the items on the checklist. Please modify your text to include the missing information. If you are certain that an item does not apply, please write "n/a" and provide a short explanation.

Upload your completed checklist as an extra file when you submit to a journal.

In your methods section, say that you used the STROBE cross sectionalreporting guidelines, and cite them as:

von Elm E, Altman DG, Egger M, Pocock SJ, Gotzsche PC, Vandenbroucke JP. The Strengthening the Reporting of Observational Studies in Epidemiology (STROBE) Statement: guidelines for reporting observational studies.

		Reporting Item	Page Number
**Title and abstract**			
Title	#1a	Indicate the study's design with a commonly used term in the title or the abstract	2,3
Abstract	#1b	Provide in the abstract an informative and balanced summary of what was done and what was found	3
**Introduction**			
Background / rationale	#2	Explain the scientific background and rationale for the investigation being reported	4
Objectives	#3	State specific objectives, including any prespecified hypotheses	4
**Methods**			
Study design	#4	Present key elements of study design early in the paper	5
Setting	#5	Describe the setting, locations, and relevant dates, including periods of	5-6

For peer review only

		recruitment, exposure, follow-up, and data collection	
Eligibility criteria	#6a	Give the eligibility criteria, and the sources and methods of selection of participants.	5-6
	#7	Clearly define all outcomes, exposures, predictors, potential confounders, and effect modifiers. Give diagnostic criteria, if applicable	5-6
Data sources / measurement	#8	For each variable of interest give sources of data and details of methods of assessment (measurement). Describe comparability of assessment methods if there is more than one group. Give information separately for for exposed and unexposed groups if applicable.	5-6
Bias	#9	Describe any efforts to address potential sources of bias	6-7
Study size	#10	Explain how the study size was arrived at	8
Quantitative variables	#11	Explain how quantitative variables were handled in the analyses. If applicable, describe which groupings were chosen, and why	6-7
Statistical methods	#12a	Describe all statistical methods, including those used to control for confounding	6-7
Statistical methods	#12b	Describe any methods used to examine subgroups and interactions	6-7
Statistical methods	#12c	Explain how missing data were addressed	6-7
Statistical methods	#12d	If applicable, describe analytical methods taking account of sampling strategy	N/A
Statistical methods	#12e	Describe any sensitivity analyses	9
**Results**			
Participants	#13a	Report numbers of individuals at each stage of study—eg numbers potentially eligible, examined for eligibility, confirmed eligible, included in the study, completing follow-up, and analysed. Give information separately for for exposed and unexposed groups if applicable.	8
Participants	#13b	Give reasons for non-participation at each stage	N/A
Participants	#13c	Consider use of a flow diagram	Figure 1

For peer review only

Descriptive data	#14a	Give characteristics of study participants (eg demographic, clinical, social) and information on exposures and potential confounders. Give information separately for exposed and unexposed groups if applicable.	8
Descriptive data	#14b	Indicate number of participants with missing data for each variable of interest	8
Outcome data	#15	Report numbers of outcome events or summary measures. Give information separately for exposed and unexposed groups if applicable.	8-9
Main results	#16a	Give unadjusted estimates and, if applicable, confounder-adjusted estimates and their precision (eg, 95% confidence interval). Make clear which confounders were adjusted for and why they were included	8-9
Main results	#16b	Report category boundaries when continuous variables were categorized	6
Main results	#16c	If relevant, consider translating estimates of relative risk into absolute risk for a meaningful time period	N/A
Other analyses	#17	Report other analyses done—e.g., analyses of subgroups and interactions, and sensitivity analyses	9
**Discussion**			
Key results	#18	Summarise key results with reference to study objectives	9
Limitations	#19	Discuss limitations of the study, taking into account sources of potential bias or imprecision. Discuss both direction and magnitude of any potential bias.	12-13
Interpretation	#20	Give a cautious overall interpretation considering objectives, limitations, multiplicity of analyses, results from similar studies, and other relevant evidence.	14
Generalisability	#21	Discuss the generalisability (external validity) of the study results	12
**Other Information**			
Funding	#22	Give the source of funding and the role of the funders for the present study and, if applicable, for the original study on which the present article is based	14-15

The STROBE checklist is distributed under the terms of the Creative Commons Attribution License CC-BY.
This checklist was completed on 07. May 2022 using https://www.goodreports.org/, a tool made by the EQUATOR Network in collaboration with Penelope.ai

For peer review only

BMJ Open

Association between obstructive sleep apnoea and cancer: a cross-sectional, population-based study of the DISCOVERY cohort

Journal:	*BMJ Open*
Manuscript ID	bmjopen-2022-064501.R1
Article Type:	Original research
Date Submitted by the Author:	20-Dec-2022
Complete List of Authors:	Palm, Andreas; Uppsala University, Department of Medical Sciences, Lung,- Allergy-, and Sleep Research; Region of Gävleborg Gävle Hospital, Centre for Research and Development<br>Theorell-Haglöw, J; Uppsala University Department of Medical Sciences, Lung,- Allergy-, and Sleep Research<br>Isakson, Johan; Region of Gävleborg Gävle Hospital<br>Ljunggren, Mirjam; Uppsala University Department of Medical Sciences, Lung,- Allergy-, and Sleep Research<br>Sundh, Josefin; Örebro University Faculty of Medicine and Health<br>Ekström, Magnus; Lunds Universitet<br>Grote, Ludger; Goteborgs Universitet, Internal Medicine and Clinical Nutrition
**Primary Subject Heading**:	Oncology
Secondary Subject Heading:	Respiratory medicine
Keywords:	SLEEP MEDICINE, ONCOLOGY, Adult thoracic medicine < THORACIC MEDICINE

SCHOLARONE™ Manuscripts

I, the Submitting Author has the right to grant and does grant on behalf of all authors of the Work (as defined in the below author licence), an exclusive licence and/or a non-exclusive licence for contributions from authors who are: i) UK Crown employees; ii) where BMJ has agreed a CC-BY licence shall apply, and/or iii) in accordance with the terms applicable for US Federal Government officers or employees acting as part of their official duties; on a worldwide, perpetual, irrevocable, royalty-free basis to BMJ Publishing Group Ltd ("BMJ") its licensees and where the relevant Journal is co-owned by BMJ to the co-owners of the Journal, to publish the Work in this journal and any other BMJ products and to exploit all rights, as set out in our licence.

The Submitting Author accepts and understands that any supply made under these terms is made by BMJ to the Submitting Author unless you are acting as an employee on behalf of your employer or a postgraduate student of an affiliated institution which is paying any applicable article publishing charge ("APC") for Open Access articles. Where the Submitting Author wishes to make the Work available on an Open Access basis (and intends to pay the relevant APC), the terms of reuse of such Open Access shall be governed by a Creative Commons licence – details of these licences and which Creative Commons licence will apply to this Work are set out in our licence referred to above.

Other than as permitted in any relevant BMJ Author's Self Archiving Policies, I confirm this Work has not been accepted for publication elsewhere, is not being considered for publication elsewhere and does not duplicate material already published. I confirm all authors consent to publication of this Work and authorise the granting of this licence.

For peer review only

Association between obstructive sleep apnoea and cancer: a cross-sectional, population-based study of the DISCOVERY cohort

Andreas Palm, MD PhD[1,2], Jenny Theorell-Haglöw PhD[1], Johan Isakson MD[2], Mirjam Ljunggren MD PhD[1], Josefin Sundh MD PhD[3], Magnus Ekström MD PhD[4], Ludger Grote MD PhD[5,6]

[1]Department of Medical Sciences, Respiratory, Allergy and Sleep Research, Uppsala University, Box 256, SE-751 05, Uppsala, Sweden

[2]Centre for Research and Development, Uppsala University, Region of Gävleborg, Gävle Hospital, SE-80188, Gävle, Sweden

[3]Department of Respiratory Medicine, Faculty of Medicine and Health, Örebro University, Örebro, Sweden

[4]Department of Clinical Sciences, Respiratory Medicine and Allergology, Faculty of Medicine, Lund University, 221 84 Lund, Sweden

[5]Centre for Sleep and Wake Disorders, Sahlgrenska Academy, Gothenburg University, 413 90 Gothenburg, Sweden

[6]Sleep Disorders Center, Department of Pulmonary Medicine, Sahlgrenska University Hospital, Gothenburg, Sweden

Correspondence to:

Andreas Palm

andreas.palm@medsci.uu.se

Keywords: OSA, cancer, hypoxia, register

For peer review only

For peer review only

Abstract

Objectives Nocturnal hypoxia in obstructive sleep apnoea (OSA) is a potential risk factor for cancer. We aimed to investigate the association between OSA measures and cancer prevalence in a large national patient cohort.

Design Cross-sectional study.

Settings 44 sleep centres in Sweden.

Participants 62,811 patients from the Swedish registry for positive airway pressure (PAP) treatment in OSA, linked to the national cancer registry and national socio-economic data (the DISCOVERY cohort).

Outcome measures After propensity score matching for relevant confounders (anthropometric data, comorbidities, socio-economic status, smoking prevalence), sleep apnoea severity, measured as apnoea-hypopnoea index (AHI) or oxygen desaturation index (ODI), were compared between those with and without cancer diagnosis up to 5 years prior to PAP initiation. Subgroup analysis for cancer subtype was performed.

Results OSA patients with cancer (n=2,093) (29.8% females, age 65.3 (SD 10.1) years, BMI 30 (interquartile range 27-34) kg/m^2) had higher median AHI (n/h) (32(IQR 20-50) versus 30 (IQR 19-45), n/h, p=0.002) and median ODI (n/h) (28 (IQR 17-46) versus 26 (IQR 16-41), p<0.001) when compared with matched OSA patients without cancer. In subgroup analysis ODI was significantly higher in OSA patients with lung cancer (N=57; 38 (21-61) versus 27 (16-43), p=0.012)), prostate cancer (N=617; 28 (17-46) versus 24 (16-39), p=0.005), and malignant melanoma (N=170; 32 (17-46) versus 25 (14-41), p=0.015).

For peer review only

Conclusions OSA mediated intermittent hypoxia was independently associated with cancer prevalence in this large, national cohort. Future longitudinal studies are warranted to study the potential protective influence of OSA treatment on cancer incidence.

Strengths and limitations of this study

- The size of the cohort with a large number of patients with verified cancer diagnosis and concomitant obstructive sleep apnoea (OSA).
- High external validity since the Swedevox registry reflects almost 90% of new continuous positive airway pressure treatment starts in Sweden initiated by 44 sleep centers across the country and the cancer registry has almost 100 % coverage.
- High internal validity since the Swedevox registry has been validated against medical record data entries and showed more than 95% correctness for age, body-mass index, and apnoea-hypopnoea index values.
- Important lifestyle risk factors for cancer including smoking, physical activity or food preferences have not been captured on an individual level in our study, but we included data on smoking prevalence in the different regions of Sweden to capture the potential influence of smoking on cancer prevalence.
- Due to the cross-sectional design, our study cannot inform about the potential causality in the association between OSA and cancer.

For peer review only

INTRODUCTION

Patients with obstructive sleep apnoea (OSA) have recurring airway collapses during sleep with corresponding episodes of hypoxia. Animal models suggest an association between intermittent hypoxia and increased tumor growth, increased angiogenesis, changes in immune function, and increases in inflammation and oxidative stress (1). Several studies investigated the potential causal relationship between OSA and cancer (2-10) and some but not all report an increased prevalence of cancer in the OSA population studied. A recent meta-analysis provided evidence for a 40-50% increased risk ratio for cancer incidence in OSA patients (11).

As OSA is associated with several other risk factors for cancer diseases, such as obesity, cardiometabolic disease, and lifestyle factors, the independent influence of OSA-related hypoxia for an increased cancer risk remained unsolved. The validity of existing data may be in part hampered by the relatively low number of reported cancer cases, a potential reporting bias due to patient-reported cancer diagnosis without the validation of independent sources, as well as by a selection bias due to single-center studies.

In Sweden, 85% of patients with OSA receiving continuous positive airway pressure (CPAP) are reported to the Swedevox registry (12, 13) and almost all patients diagnosed with cancer are reported to the mandatory governmental Cancer Registry (14). The Swedish system with personal identity numbers (PIN) (15) allows for cross-linkage of various registries, creating large databases with reliable data quality. Thereby, our current study aimed to overcome some of the limitations mentioned above by exploring the association between measures of OSA and cancer prevalence in a large, multicentric national cohort of OSA patients referred

For peer review only

for PAP treatment using validated data from the national cancer registry for verified cancer diagnosis and subclassification.

For peer review only

METHODS

Study design and population

This was a national, population-based, cross-sectional study from the "course of DIsease in patients reported to Swedish CPAP, Oxygen and VEntilator RegistrY" (DISCOVERY) cohort. The study has been detailed elsewhere (13). In this study, data on patients aged ≥ 16 years with OSA on CPAP therapy, correctly and prospectively reported to the Swedevox registry (12) between 1st of July 2010 and 31th December 2017 were cross-linked with data from the mandatory National cancer registry (16) for information about cancer diagnosis, the Prescribed Drug Registry with information about all prescriptions of medications (17) and the National Patient Registry (NPR) covering data and diagnosis from all inpatient and outpatient visits at governmental hospitals (18). Socio-economic data (marital status, education level) were covered from Statistics Sweden (19). An overview of the study flow is presented in Figure 1.

End-point variables and co-variables

The primary variable of interest was captured by the diagnose codes "all-cause cancer" (ICD-10 code C00-99) up to five years prior initiation of CPAP therapy. Additional information was captured from organ specific cancer: Ear-, neck-, and throat cancer (ENT) (C1-14), colon (C18), lung (C34), malignant melanomas (C43), breast (C50), prostate (C61), urinary (C66-67) and malignant brain tumors (C71). Information on "Skin cancer" (C44) other than malign melanoma was excluded.

Information on sex, body-mass index (BMI), apnoea-hypopnoea index (AHI), oxygen desaturation index (ODI), Epworth sleepiness scale score (ESS), and date of initiation of

For peer review only

treatment were derived from the Swedevox registry (12, 13). Comorbid heart failure (ICD-10 codes I11, I42 and I50) and ischemic heart disease (ICD-10 code I20-25) was derived from NPR (18). Comorbid obstructive lung disease (OLD) was classified according to accepted practice by ≥ 2 collections of anti-obstructive drugs (ATC code R03) ≤12 months prior to initiation of CPAP and comorbid diabetes was classified by ≥1 collection of antidiabetic drugs (A10) ≤6 months prior initiation of CPAP therapy was registered in the national Prescribed Drug Registry (17). Information on educational level was obtained from Statistic Sweden and was categorized as low (<=9 years), medium (10-12 years), or high (≥13 years), corresponding to compulsory school, secondary school, and post-secondary school (college and university), respectively. The Swedevox registry does not contain information about individual smoking data. A marker for smoking exposure was generated from information on annual smoking prevalence between 2006 and 2018, in each county according to Statistics Sweden's Living Conditions Surveys (ULF/ SILC) (20). Counties were trichotomized according to mean smoking rate (8.3-12.0%, 12.1-13.5%, and 13.6-14.8%).

Statistical analyses

OSA severity was graded by the AHI and ODI both as continuous and categorical variables (AH! <5, 5- <15 (mild OSA), 15-<30 (moderate OSA), and ≥30 (severe OSA)). Clinical cut-off values for ODI severity are not defined, and we, therefore, classified ODI into quartiles when used as a categorical variable. Association between sleep recording variables and all-cause cancer diagnosis ≤5 years prior to the initiation of CPAP therapy was analyzed. In detail, the relationship between prevalent all-cause cancer and organ-specific cancer and AHI and ODI were analyzed using propensity score matched models (21). For propensity score matching, independent co-variables were used as anthropometrics (age, sex, BMI), presence of

For peer review only

comorbidities at baseline (ischemic heart disease, heart failure, diabetes, OLD), socio-economic data (civil status and educational level), start year of CPAP treatment and smoking prevalence (by county, trichotomized). These factors were chosen based on direct acyclic graphs using the browser-based environment DAGitty (www. dagitty.net) [22]. In addition, we performed a parallel analysis applying logistic regression analysis in the entire baseline population to predict “all-cause cancer diagnosis” independent of all mentioned covariates used for the propensity matching procedure.

Normally distributed continuous data were expressed as mean ± SD, and skewed distributed data were expressed as median with interquartile range (IQR). Categorical data were presented as frequencies and percentages. Differences between groups were analyzed using chi-2 test for categorical variables and Student´s t-test for continuous variables. A p-value <0.05 was considered statistically significant. Statistical analyses were conducted using the software packages Stata, version 17.0 (StataCorp LP; College Station, TX 77845 USA).

Patient and public involvement

None.

For peer review only

RESULTS

Patient characteristics

Clinical data, in particular age, and frequency of comorbidities varied significantly between unmatched cases (N=2,933) and controls (N=59,878). The final propensity score-matched study population consisted of 2,096 patients with cancer (29.8% females, mean age 65.3 (SD 10.1) years, median BMI 30 (IQR 27-34) kg/m^2) and 2,096 matched OSA patients without cancer (**Table 1**). Totally in the cohort, 91 patients had lung cancer, 46 ear-nose-throat cancer, 798 prostate cancer, 239 breast cancer, 191 colon cancer, 134 urinary cancer, 254 malignant melanoma, and 16 malignant brain tumors ≤ 5 years prior to CPAP initiation.

Table 1. Characteristics of unmatched and propensity score matched study population

	Unmatched		Matched	
	No cancer N=59,878	All-cause cancer N=2,933	No cancer N=2,093	All-cause cancer N=2,093
Sex, females (%)	17,647 (29.5%)	903 (30.8%)	661 (31.6%)	623 (29.8%)
Age, years	56.8 (12.5)	65.5 (10.2)	65.3 (10.2)	65.3 (10.1)
BMI, kg/m2	31 (28-35)	30 (27-34)	30 (27-34)	30 (27-34)
ESS, units	10.3 (5.0)	9.9 (4.9)	9.6 (4.9)	10.0 (4.9)
Ischemic heart disease	5,671 (9.5%)	425 (14.5%)	311 (14.9%)	309 (14.8%)
Heart failure	3,263 (5.4%)	277 (9.4%)	180 (8.6%)	186 (8.9%)
Diabetes mellitus	8,548 (14.3%)	526 (17.9%)	360 (17.2%)	366 (17.5%)
OLD	7,610 (12.7%)	462 (15.8%)	325 (15.5%)	327 (15.6%)
Civil status				
Married	31,926 (53.5%)	1,771 (61.0%)	1,300 (62.1%)	1,280 (61.2%)
Unmarried	14,675 (24.6%)	392 (13.5%)	289 (13.8%)	275 (13.1%)
Divorced	10,561 (17.7%)	520 (17.9%)	340 (16.2%)	378 (18.1%)
Widower/widow	2,529 (4.2%)	219 (7.5%)	164 (7.8%)	160 (7.6%)
Level of education				
Low (≤9 years)	10,547 (21.7%)	605 (26.1%)	540 (25.8%)	546 (26.1%)
Medium (10-12 years)	25,065 (51.5%)	1,061 (45.8%)	977 (46.7%)	959 (45.8%)

For peer review only

High (≥13 years)	13,060 (26.8%)	653 (28.2%)	576 (27.5%)	588 (28.1%)

Matched by anthropometric variables (sex, age, BMI), comorbidities at baseline (ischemic heart disease, heart failure, diabetes mellitus and obstructive lung disease), socioeconomic factors (civil status and educational level), start year of CPAP treatment and county trichotomized by smoking prevalence. Data are presented as mean (SD) or median (IQR) for continuous measures, and n (%) for categorical measures. AHI: apnea-hypopnoea index; BMI: body-mass index; ESS: Epworth sleepiness scale; ODI: oxygen desaturation index; OLD: obstructive lung disease.

After matching procedure, clinical characteristics did not differ between cases and controls except for the Epworth Sleepiness Scale score which was slightly elevated in cases compared with controls (ESS score 10.0 (SD 4.9) versus 9.6 (SD 4.9), p=0.02, respectively).

Sleep apnoea severity in OSA patients with and without cancer

Cases with cancer disease had higher AHI (median 32 (IQR 20-50) versus 30 (19-45), n/h, p=0.002) and ODI (28 (17-46) versus 26 (16-41), p<0.001) when compared with matched controls without cancer (**Table 2** and **Figure 2**). Severe nocturnal intermittent hypoxia (ODI quartile IV (>45n/h)) was significantly more prevalent in cases (25.7%) compared to controls (20.8%), p=0.001 for ODI categories. Corresponding numbers for the highest AHI class (≥30 n/h) were 54.9% in cases and 52.4% in controls but the differences in AHI categories did not reach statistical significance.

Subgroup analysis confirmed that ODI was significantly higher in OSA patients with lung cancer (N=57, median 38 (IQR 21-61) versus 27 (16-43), p=0.012)), prostate cancer (N=617, 28 (17-46) versus 24 (16-39), p=0.005), and malignant melanoma (N=170, 32 (17-46) versus 25 (14-41), p=0.015) when compared with corresponding OSA patients without cancer (**Table 3**). The remaining cancer subtypes did not differ in OSA severity measures.

For peer review only

Table 2. OSA severity in propensity score-matched patients with OSA stratified by the presence of cancer diagnosis ≤ 5 years prior to initiation of CPAP treatment

	No cancer N=2,093	All-cause cancer N=2,093	p-value
AHI, events/hour	30 (19-45)	32 (20-50)	0.002
AHI category, events/hour			0.19
AHI <5	15 (0.7%)	14 (0.7%)	
AHI 5-14.9	272 (13.0%)	247 (11.8%)	
AHI 15-29.9	718 (34.3%)	682 (32.6%)	
AHI ≥30	1,088 (52.0%)	1,150 (54.9%)	
ODI, events/hour	26 (16-41)	28 (17-46)	<0.001
ODI, quartiles, events/hour			0.002
ODI 0-15.9	501 (23.9%)	433 (20.7%)	
ODI 16-27.9	587 (28.0%)	576 (27.5%)	
ODI 28-44.9	569 (27.2%)	546 (26.1%)	
ODI ≥45	436 (20.8%)	538 (25.7%)	

Matched by anthropometric variables (sex, age, BMI), comorbidities at baseline (ischemic heart disease, heart failure, diabetes mellitus, and obstructive lung disease), socioeconomic factors (civil status and educational level), start year of CPAP treatment and county trichotomized by smoking prevalence. Data are presented as median (IQR) for continuous measures, and n (%) for categorical measures. AHI: apnea-hypopnoea index; ODI: oxygen desaturation index; OSA: obstructive sleep apnoea.

For peer review only

Table 3. Frequency of organ-specific cancer prevalence in patients with OSA ≤5 years prior to the initiation of CPAP in relation to AHI and ODI in propensity score matched cohorts

For peer review only

	No lung cancer N=57	Lung cancer N=57	p-value	No ENT cancer N=31	ENT cancer N=31	p-value	No prostate cancer N=617	Prostate cancer N=617	p-value
AHI, events/hour	28 (18-44)	38 (25-56)	0.019	29 (18-46)	30 (17-41)	0.30	28 (19-44)	33 (20-50)	0.006
AHI category, events/hour			0.10			0.036			0.035
AHI <5	0	0		0	0		4 (0.6%)	2 (0.3%)	
AHI 5-14.9	8 (14.0%)	6 (10.5%)		1 (3.2%)	7 (22.6%)		70 (11.3%)	66 (10.7%)	
AHI 15-29.9	21 (36.8%)	12 (21.1%)		15 (48.4%)	8 (25.8%)		251 (40.7%)	208 (33.7%)	
AHI >30	28 (49.1%)	39 (68.4%)		15 (48.4%)	16 (51.6%)		292 (47.3%)	341 (55.3%)	
ODI, events/hour	27 (16-43)	38 (21-61)	0.012	24 (15-46)	23 (13-39)	0.22	24 (16-39)	28 (17-46)	0.005
ODI, quartiles, events/hour			0.034			0.21			0.009
ODI 0-15.9	13 (22.8%)	6 (10.5%)		8 (25.8%)	11 (35.5%)		147 (23.8%)	129 (20.9%)	
ODI 16-27.9	17 (29.8%)	17 (29.8%)		10 (32.3%)	6 (19.4%)		210 (34.0%)	170 (27.6%)	
ODI 28-44.9	15 (26.3%)	9 (15.8%)		5 (16.1%)	10 (32.3%)		139 (22.5%)	161 (26.1%)	
ODI >45	12 (21.1%)	25 (43.9%)		8 (25.8%)	4 (12.9%)		121 (19.6%)	157 (25.4%)	
	No Breast cancer N=162	Breast cancer N=162	p-value	No colon cancer N=123	Colon cancer N=123	p-value	No urinary cancer N=93	Urinary cancer N=93	p-value
AHI, events/hour	28 (17-46)	28 (18-46)	0.49	33 (21-53)	30 (21-48)	0.41	31 (19-47)	35 (24-52)	0.15
AHI category, events/hour			0.59			0.31			0.40
AHI <5	4 (2.5%)	2 (1.2%)		4 (3.3%)	2 (1.6%)		1 (1.1%)	1 (1.1%)	
AHI 5-14.9	29 (17.9%)	24 (14.8%)		10 (8.1%)	13 (10.6%)		13 (14.0%)	6 (6.5%)	
AHI 15-29.9	51 (31.5%)	60 (37.0%)		35 (28.5%)	46 (37.4%)		26 (28.0%)	30 (32.3%)	
AHI >30	78 (48.1%)	76 (46.9%)		74 (60.2%)	62 (50.4%)		53 (57.0%)	56 (60.2%)	
ODI, events/hour	24 (14-39)	27 (17-49)	0.26	28 (18-50)	26 (17-43)	0.47	28 (16-47)	32 (21-50)	0.11

For peer review only

ODI, quartiles, events/hour			0.30			0.71			0.67
ODI 0-15.9	49 (30.2%)	38 (23.5%)		24 (19.5%)	26 (21.1%)		20 (21.5%)	14 (15.1%)	
ODI 16-27.9	39 (24.1%)	49 (30.2%)		34 (27.6%)	36 (29.3%)		26 (28.0%)	25 (26.9%)	
ODI 28-44.9	40 (24.7%)	34 (21.0%)		28 (22.8%)	32 (26.0%)		22 (23.7%)	25 (26.9%)	
ODI >45	34 (21.0%)	41 (25.3%)		37 (30.1%)	29 (23.6%)		25 (26.9%)	29 (31.2%)	

	No malignant melanoma N=170	Malignat melanoma N=170	p-value	No malignat brain tumor N=8	Malignant brain tumor N=8	p-value
AHI, events/hour	28 (17-45)	35 (23-52)	0.003	31 (12-40)	40 (16-54)	0.25
AHI category, events/hour			0.072			0.86
AHI <5	2 (1.2%)	1 (0.6%)		0	0	
AHI 5-14.9	25 (14.7%)	15 (8.8%)		2 (25.0%)	2 (25.0%)	
AHI 15-29.9	65 (38.2%)	53 (31.2%)		1 (12.5%)	1 (12.5%)	
AHI >30	78 (45.9%)	101 (59.4%)		5 (62.5%)	5 (62.5%)	
ODI, events/hour	25 (14-41)	32 (17-46)	0.015	29 (13-34)	36 (15-56)	0.19
ODI, quartiles, events/hour			0.13			0.28
ODI 0-15.9	48 (28.2%)	32 (18.8%)		3 (37.5%)	2 (25.0%)	
ODI 16-27.9	47 (27.6%)	45 (26.5%)		1 (12.5%)	1 (12.5%)	
ODI 28-44.9	44 (25.9%)	50 (29.4%)		4 (50.0%)	2 (25.0%)	
ODI >45	31 (18.2%)	43 (25.3%)		0 (0.0%)	3 (37.5%)	

For peer review only

For peer review only - http://bmjopen.bmj.com/site/about/guidelines.xhtml

Matched by anthropometric variables (sex, age, BMI), comorbidities at baseline (ischemic heart disease, heart failure, diabetes mellitus, and obstructive lung disease), socioeconomic factors (civil status and educational level), start year of CPAP treatment and county trichotomized by smoking prevalence. AHI and ODI are expressed as both continuous and categorized variables. Data are presented as median (IQR) for continuous measures, and n (%) for categorical measures. AHI: apnea-hypopnoea index; CPAP: continuous positive airway pressure; ENT: ear-, nose-, and throat; ODI: oxygen desaturation index; OSA: obstructive sleep apnoea.

For peer review only

In a parallel analysis including the entire cohort (N=62,811), multivariable logistic regression analysis with cancer as the independent variable, and adjustment for the same variables as in the propensity score matching, we identified significant odds ratios for ODI and AHI (1.05 (1.03-1.07) and 1.04 (1.02-1.07) per 10 units, p<0.001, respectively) confirming the data obtained by propensity score matching.

For peer review only

Discussion

Main findings

Our study identified several important findings. First, OSA is associated with cancer prevalence independent of a substantial number of important confounders. The added risk is small and predominantly seen in patients with severe intermittent hypoxia during sleep measured by ODI rather than in those with frequent respiratory events during sleep measured by AHI. Second, the association between intermittent hypoxia and cancer was identified in lung cancer, malignant melanoma, and prostate cancer. Our study underlines the importance of further studies to investigate if preventing and treating sleep apnoea may have beneficial effects on cancer incidence and any outcomes of cancer treatment.

Cancer prevalence in other cohort studies

Results from previous studies on OSA and cancer are conflicting. One early Spanish multi-center study (4,910 patients) identified an increased cancer incidence in OSA patients with an overall odds ratio of 1.45 (95% CI 1.1-1.9) (2). In a Canadian cohort study (n= 10,149) from a single sleep clinic at an academic hospital, 520 patients had a cancer diagnosis at baseline

For peer review only - http://bmjopen.bmj.com/site/about/guidelines.xhtml

and 627 patients of the cancer-free patients at baseline were reported with incident cancer at follow-up (7.8 years) (10). No association between the severity of OSA and cancer was found for prevalent or incident cancer. In a twice as large European cross-sectional study (n=19,556 of whom 388 self-reported cancer diagnosis at the time of sleep study), an association between AHI and ODI was found in women but not in men (8). In a recent French cohort study (n=8,748, 5.8-year follow-up, 718 incident cancer cases), an association between nocturnal hypoxia, defined as the percent of the night spent with saturation <90%, and incident cancer was found while there were no associations between AHI or ODI and incident cancer after adjustment for confounders (9). A recent meta-analysis of 12 studies, including 184,915 patients with OSA (n=75,367 and 3,805 cancer cases) and patients without OSA (n=109,548 and 2,110 cancer cases) identified a dose-dependent increased risk of incident all-cause cancer with an odds ratio of 1.14 (95% CI: 1.04-1.25, p=0.006) for mild OSA, 1.36 (1.32-1.92;p <0.001) for moderate OSA and 1.59 (95% CI: 1.45-1.74; P < 0.001) for severe OSA (11). Unfortunately, this meta-analysis did not evaluate the association between the amount of OSA-related hypoxic measures and cancer incidence. In this context, our cross-sectional data in one of the largest OSA patient populations so far is in line with previous findings. OSA appears to be associated with cancer, but effect sizes are generally small, and large cohorts are needed to detect any independent effect of OSA on cancer incidence. It cannot be excluded that other confounders, not yet considered in any of the published analyses, may further explain part of the effect sizes accounted for OSA. Notably, several studies show that OSA measures reflecting hypoxic burden appear to better predict the risk for cancer when compared with AHI as traditional OSA severity marker (8, 9). Due to the exact classification of cancer type through the Swedish national registry, we could perform subtype analysis on the influence of OSA-related cancers. We identified more

For peer review only - http://bmjopen.bmj.com/site/about/guidelines.xhtml

severe OSA in lung cancer, malignant melanoma and in prostate cancer which is in line with previous study results (23-26). In contrast, we did not identify an increased prevalence of OSA in patients with breast cancer (27, 28) .

Potential mechanisms between the association of OSA and cancer

Previous studies have explored potential mechanistic pathways for the association between OSA and cancer. Experimental studies mimicking OSA by using an intermittent hypoxia model show that intermittent hypoxia promotes incident tumor cell transition, tumor growth, and metastasis. Several in vitro cell models identified intermittent hypoxia to promote tumor cell growth in different cell lines (29). In vivo mouse models showed that melanoma metastasis was increased in animals exposed to intermittent hypoxia mimicking severe OSA (30). Although these data suggest a possible OSA-related mechanistic pathway for cancer growth and aggressiveness, further studies are needed to explore if and how OSA promote the initial occurrence of cancer. In this context, obesity is an important confounder of the association between OSA and cancer. Obesity in the general population has nearly doubled in the last 20 years and by 2017, 17% of the Swedish adult population are obese, defined as BMI exceeding 30 kg/m^2 (31). Presence of OSA is closely linked to obesity (32), and in Sweden, the mean BMI when initiating continuous positive airway pressure (CPAP) therapy is 32 kg/m^2 (12). Several cancer forms, i.e. breast-, colon-, kidney-, pancreas-, and esophageal cancer, are also associated with higher BMI (33). As both age-standardized cancer incidence (34) as well as the prevalence of obesity are increasing (31) in parallel, we carefully controlled for both factors in our analysis.

For peer review only - http://bmjopen.bmj.com/site/about/guidelines.xhtml

Inflammation may be considered as a third potential pathway for the link between OSA and cancer. OSA increases systemic inflammation with increased levels of hsCRP, IL-6 and TNF-α (35, 36). Systemic inflammation has been linked to the risk of cancer/cancer development but also tumor growth (37). In addition, a low degree of inflammation has been identified in adipocytes which may contribute to the overall increased inflammation in obesity. Further studies linking markers of inflammation to cancer incidence in OSA populations are of particular interest.

Our study has several strengths. First, the study has a high validity as we included a large number of patients with a verified cancer diagnosis and concomitant OSA. The National cancer registry has the highest quality due to regularly performed validation studies by the National Social Welfare Board. The sleep apnoea-related data within the Swedevox registry has recently been validated against the medical record data entries and showed more than 95% correctness for age, BMI, and AHI values (38). Second, our study cohort is representative for studying the association between OSA and cancer prevalence. The Swedevox registry reflects almost 90% of new CPAP treatment starts in Sweden initiated by 44 sleep centers across the country (12). The national cancer registry has nearly 100% coverage (16). Socio-economic data are based on information from the National Tax Agency. No patient was lost due to missing data in the national registries. Third, we used a strong analysis methodology with sufficient power due to a large number of cases. The propensity score matching of cases and controls adjusting for many important confounders can be viewed as close to a randomized study setting. A parallel multivariable Cox-regression analysis confirmed the results of the propensity score matched analysis.

Nonetheless study limitations need also to be addressed. First, sleep apnoea within the Swedevox registry is almost exclusively classified by polygraphy and not by

For peer review only - http://bmjopen.bmj.com/site/about/guidelines.xhtml

polysomnography. However, device methodology is very homogenous in Sweden and national guidelines for the analysis of polygraphic recordings are published (39). Polygraphic recording may underestimate the actual AHI classification but to a lesser extent, the ODI count. This may, in part explain why ODI is a stronger predictor of cancer prevalence than AHI. Second, we do not have other measures of nocturnal hypoxia other than ODI, for example, the time spent in saturation below 90% or hypoxic burden analysis of the saturation curve, recently described as a significant predictor of cancer in OSA patients (2, 8, 9, 40). Third, important lifestyle risk factors for cancer, including smoking, physical activity, or food preferences, have not been captured on an individual level in our study. However, we have a strong classification of socio-economic factors. We also included data on smoking prevalence in the different regions of Sweden to capture the potential influence of smoking on cancer prevalence. Research into the impact of socioeconomic factors on cancer incidence typically find a larger prevalence of solid tumors in groups with less fortunate social standing with breast cancer being the sole outlier. The single most important variable in cancer incidence is tobacco smoking where large differences are seen between socioeconomic groups (41). In case of lacking information on individual patient data for such risk factors, the use of socioeconomic stratification represents the best available method to adjust for this. Fourth, our study included only PAP-treated OSA patients reflecting the upper range of OSA severity and not the entire OSA spectrum. This patient selection may have limited our possibility to fully assess the dose response relationship between OSA severity and cancer prevalence. However, the OSA patient group with severe intermittent hypoxia demonstrated the strongest risk increase for cancer prevalence. Finally, due to the cross-sectional study design, our study cannot speculate about the causality in the association between OSA and cancer.

For peer review only - http://bmjopen.bmj.com/site/about/guidelines.xhtml

Clinical implications and research agenda

Our study provides further cross-sectional evidence to the important clinical question on the association between OSA and cancer in OSA patients. Even if we did not analyze longitudinal data on the incidence of cancer, we see a significant yet limited effect size for OSA on the risk for prevalent cancer. More importantly, future analysis needs to focus on the effect of OSA treatment with PAP on cancer incidence and survival.

CONCLUSION

OSA mediated intermittent hypoxia is an independent risk factor for cancer in this large, national OSA patient cohort. Future longitudinal studies are warranted to study the potential influence of OSA treatment on cancer incidence in patients with OSA.

Funding

AP was supported by grants from Gävle Cancer Foundation (grant number N/A), the Swedish Society for Sleep Research and Sleep Medicine (grant number N/A), Centre for Research and Development, Uppsala University/Region Gävleborg (grant number N/A), Bror Hjerpstedt´s Foundation (grant number N/A) and Uppsala Heart and Lung Foundation (grant number N/A) and Regional Research Council in Mid Sweden (RFR-931234). JTH was supported by a grant from Swedish Heart and Lung Foundation (20190607, 20190611). ME was supported by unrestricted grants from the Swedish Research Council (Dnr 2019-02081). LG was supported by the Swedish Heart and Lung Foundation (20180567, 20210529).

For peer review only - http://bmjopen.bmj.com/site/about/guidelines.xhtml

Competing interests

No conflicts of interest exist for the authors regarding the submitted manuscript.

Data availability statement

The steering committee of the Swedevox quality registry will consider reasonable requests for the sharing of deidentified patient-level data. Requests should be made to the corresponding author.

Contributors

AP, JTH, JI, ML, JS, ME and LG contributed to the conception and design of the study. AP performed statistical analyses, and all authors verified the underlying data. AP and LG wrote the first draft. All authors had full access to all the data in the study and had final responsibility for the decision to submit for publication. All authors participated in data interpretation, drafting of the manuscript, and final approval for submission.

Ethics statement

The study protocol was approved by the Ethics Committee at the Medical Faculty at Lund University and the Swedish Ethical Review Authority, Dnr. 2018/51, 2019-01420, 2020-02721, 2021-04984, 2022-00745, and 2022-02012. According to Swedish law, patients entering a national quality registry like Swedevox are informed, and can withdraw from registration at any time, but do not sign an informed consent.

REFERENCES

For peer review only - http://bmjopen.bmj.com/site/about/guidelines.xhtml

For peer review only

1. Almendros I, Montserrat JM, Torres M, Bonsignore MR, Chimenti L, Navajas D, et al. Obesity and intermittent hypoxia increase tumor growth in a mouse model of sleep apnea. Sleep medicine. 2012;13(10):1254-60. Epub 2012/11/15. doi: 10.1016/j.sleep.2012.08.012. PubMed PMID: 23149216.
2. Campos-Rodriguez F, Martinez-Garcia MA, Martinez M, Duran-Cantolla J, Pena Mde L, Masdeu MJ, et al. Association between obstructive sleep apnea and cancer incidence in a large multicenter Spanish cohort. American journal of respiratory and critical care medicine. 2013;187(1):99-105. Epub 2012/11/17. doi: 10.1164/rccm.201209-1671OC. PubMed PMID: 23155146.
3. Christensen AS, Clark A, Salo P, Nymann P, Lange P, Prescott E, et al. Symptoms of sleep disordered breathing and risk of cancer: a prospective cohort study. Sleep. 2013;36(10):1429-35. Epub 2013/10/02. doi: 10.5665/sleep.3030. PubMed PMID: 24082302; PubMed Central PMCID: PMC3773192.
4. Nieto FJ, Peppard PE, Young T, Finn L, Hla KM, Farre R. Sleep-disordered breathing and cancer mortality: results from the Wisconsin Sleep Cohort Study. American journal of respiratory and critical care medicine. 2012;186(2):190-4. Epub 2012/05/23. doi: 10.1164/rccm.201201-0130OC. PubMed PMID: 22610391; PubMed Central PMCID: PMC3406081.
5. Chang WP, Liu ME, Chang WC, Yang AC, Ku YC, Pai JT, et al. Sleep apnea and the subsequent risk of breast cancer in women: a nationwide population-based cohort study. Sleep medicine. 2014;15(9):1016-20. Epub 2014/08/03. doi: 10.1016/j.sleep.2014.05.026. PubMed PMID: 25085620.
6. Chen JC, Hwang JH. Sleep apnea increased incidence of primary central nervous system cancers: a nationwide cohort study. Sleep medicine. 2014;15(7):749-54. Epub 2014/06/04. doi: 10.1016/j.sleep.2013.11.782. PubMed PMID: 24891080.
7. Gozal D, Farre R, Nieto FJ. Putative Links Between Sleep Apnea and Cancer: From Hypotheses to Evolving Evidence. Chest. 2015;148(5):1140-7. Epub 2015/05/29. doi: 10.1378/chest.15-0634. PubMed PMID: 26020135; PubMed Central PMCID: PMC4631033.
8. Pataka A, Bonsignore MR, Ryan S, Riha RL, Pepin JL, Schiza S, et al. Cancer prevalence is increased in females with sleep apnoea: data from the ESADA study. The European respiratory journal. 2019;53(6). Epub 2019/05/22. doi: 10.1183/13993003.00091-2019. PubMed PMID: 31109987.
9. Justeau G, Gervès-Pinquié C, Le Vaillant M, Trzepizur W, Meslier N, Goupil F, et al. Association Between Nocturnal Hypoxemia and Cancer Incidence in Patients Investigated for OSA: Data From a Large Multicenter French Cohort. Chest. 2020;158(6):2610-20. Epub 2020/07/07. doi: 10.1016/j.chest.2020.06.055. PubMed PMID: 32629036.
10. Kendzerska T, Leung RS, Hawker G, Tomlinson G, Gershon AS. Obstructive sleep apnea and the prevalence and incidence of cancer. CMAJ : Canadian Medical Association journal = journal de l'Association medicale canadienne. 2014;186(13):985-92. Epub 2014/08/07. doi: 10.1503/cmaj.140238. PubMed PMID: 25096668; PubMed Central PMCID: PMC4162778.
11. Cheng H, Li D. Investigation into the association between obstructive sleep apnea and incidence of all-type cancers: a systematic review and meta-analysis. Sleep medicine. 2021;88:274-81. Epub 20210604. doi: 10.1016/j.sleep.2021.05.031. PubMed PMID: 34219029.
12. The Swedevox registry. Annual report 2020. 2021 [14 Sep 2021]. Available from: https://www.ucr.uu.se/swedevox/rapporter/arsrapporter/arsrapporter/arsrapport-2020.
13. Palm A, Agren K, Grote L, Ljunggren M, Midgren B, Sundh J, et al. Course of DISease In patients reported to the Swedish CPAP Oxygen and VEntilator RegistrY (DISCOVERY) with population-based controls. BMJ Open. 2020;10(11):e040396. Epub 2021/01/15. doi: 10.1136/bmjopen-2020-040396. PubMed PMID: 33444200; PubMed Central PMCID: PMC7678356.
14. Barlow L, Westergren K, Holmberg L, Talback M. The completeness of the Swedish Cancer Register: a sample survey for year 1998. Acta oncologica (Stockholm, Sweden). 2009;48(1):27-33. Epub 2008/09/04. doi: 10.1080/02841860802247664. PubMed PMID: 18767000.

For peer review only - http://bmjopen.bmj.com/site/about/guidelines.xhtml

15. Ludvigsson JF, Otterblad-Olausson P, Pettersson BU, Ekbom A. The Swedish personal identity number: possibilities and pitfalls in healthcare and medical research. European journal of epidemiology. 2009;24(11):659-67. Epub 2009/06/09. doi: 10.1007/s10654-009-9350-y. PubMed PMID: 19504049; PubMed Central PMCID: PMC2773709.
16. Pukkala E, Engholm G, Højsgaard Schmidt LK, Storm H, Khan S, Lambe M, et al. Nordic Cancer Registries - an overview of their procedures and data comparability. Acta oncologica (Stockholm, Sweden). 2018;57(4):440-55. Epub 2017/12/12. doi: 10.1080/0284186x.2017.1407039. PubMed PMID: 29226751.
17. Wettermark B, Hammar N, Fored CM, Leimanis A, Otterblad Olausson P, Bergman U, et al. The new Swedish Prescribed Drug Register--opportunities for pharmacoepidemiological research and experience from the first six months. Pharmacoepidemiology and drug safety. 2007;16(7):726-35. Epub 2006/08/10. doi: 10.1002/pds.1294. PubMed PMID: 16897791.
18. Ludvigsson JF, Andersson E, Ekbom A, Feychting M, Kim JL, Reuterwall C, et al. External review and validation of the Swedish national inpatient register. BMC public health. 2011;11:450. Epub 2011/06/11. doi: 10.1186/1471-2458-11-450. PubMed PMID: 21658213; PubMed Central PMCID: PMC3142234.
19. Ludvigsson JF, Svedberg P, Olen O, Bruze G, Neovius M. The longitudinal integrated database for health insurance and labour market studies (LISA) and its use in medical research. European journal of epidemiology. 2019;34(4):423-37. Epub 2019/04/01. doi: 10.1007/s10654-019-00511-8. PubMed PMID: 30929112; PubMed Central PMCID: PMC6451717.
20. Living Conditions Surveys (ULF/SILC) 2019 [updated October 14, 2021February 10, 2022]. Available from: http://www.scb.se/le0101-en.
21. Austin PC. An Introduction to Propensity Score Methods for Reducing the Effects of Confounding in Observational Studies. Multivariate Behav Res. 2011;46(3):399-424. Epub 20110608. doi: 10.1080/00273171.2011.568786. PubMed PMID: 21818162; PubMed Central PMCID: PMC3144483.
22. Lederer DJ, Bell SC, Branson RD, Chalmers JD, Marshall R, Maslove DM, et al. Control of Confounding and Reporting of Results in Causal Inference Studies. Guidance for Authors from Editors of Respiratory, Sleep, and Critical Care Journals. Annals of the American Thoracic Society. 2019;16(1):22-8. Epub 2018/09/20. doi: 10.1513/AnnalsATS.201808-564PS. PubMed PMID: 30230362.
23. Cheong AJY, Tan BKJ, Teo YH, Tan NKW, Yap DWT, Sia CH, et al. Obstructive Sleep Apnea and Lung Cancer: A Systematic Review and Meta-Analysis. Annals of the American Thoracic Society. 2022;19(3):469-75. doi: 10.1513/AnnalsATS.202108-960OC. PubMed PMID: 34792438.
24. Martinez-Garcia MA, Campos-Rodriguez F, Nagore E, Martorell A, Rodriguez-Peralto JL, Riveiro-Falkenbach E, et al. Sleep-Disordered Breathing Is Independently Associated With Increased Aggressiveness of Cutaneous Melanoma: A Multicenter Observational Study in 443 Patients. Chest. 2018;154(6):1348-58. Epub 20180727. doi: 10.1016/j.chest.2018.07.015. PubMed PMID: 30059679.
25. Chung WS, Lin CL. Sleep disorders associated with risk of prostate cancer: a population-based cohort study. BMC Cancer. 2019;19(1):146. Epub 20190213. doi: 10.1186/s12885-019-5361-6. PubMed PMID: 30760242; PubMed Central PMCID: PMC6375129.
26. Lee EJ, Suh JD, Cho JH. The incidence of prostate cancer is increased in patients with obstructive sleep apnea: Results from the national insurance claim data 2007-2014. Medicine. 2021;100(6):e24659. doi: 10.1097/md.0000000000024659. PubMed PMID: 33578596.
27. Choi JH, Lee JY, Han KD, Lim YC, Cho JH. Association between obstructive sleep apnoea and breast cancer: The Korean National Health Insurance Service Data 2007-2014. Sci Rep. 2019;9(1):19044. Epub 20191213. doi: 10.1038/s41598-019-55551-7. PubMed PMID: 31836779; PubMed Central PMCID: PMC6910956.
28. Madut A, Fuchsova V, Man H, Askar S, Trivedi R, Elder E, et al. Increased prevalence of obstructive sleep apnea in women diagnosed with endometrial or breast cancer. PloS one. 2021;16(4):e0249099. Epub 20210407. doi: 10.1371/journal.pone.0249099. PubMed PMID: 33826649; PubMed Central PMCID: PMC8026058.

For peer review only - http://bmjopen.bmj.com/site/about/guidelines.xhtml

29. Bhaskara VK, Mohanam I, Rao JS, Mohanam S. Intermittent hypoxia regulates stem-like characteristics and differentiation of neuroblastoma cells. PloS one. 2012;7(2):e30905. Epub 20120217. doi: 10.1371/journal.pone.0030905. PubMed PMID: 22363512; PubMed Central PMCID: PMC3281893.
30. Li L, Ren F, Qi C, Xu L, Fang Y, Liang M, et al. Intermittent hypoxia promotes melanoma lung metastasis via oxidative stress and inflammation responses in a mouse model of obstructive sleep apnea. Respiratory research. 2018;19(1):28. Epub 20180212. doi: 10.1186/s12931-018-0727-x. PubMed PMID: 29433520; PubMed Central PMCID: PMC5809953.
31. Hemmingsson E, Ekblom Ö, Kallings LV, Andersson G, Wallin P, Söderling J, et al. Prevalence and time trends of overweight, obesity and severe obesity in 447,925 Swedish adults, 1995-2017. Scandinavian journal of public health. 2020:1403494820914802. Epub 2020/05/01. doi: 10.1177/1403494820914802. PubMed PMID: 32349623.
32. Tishler PV, Larkin EK, Schluchter MD, Redline S. Incidence of sleep-disordered breathing in an urban adult population: the relative importance of risk factors in the development of sleep-disordered breathing. JAMA : the journal of the American Medical Association. 2003;289(17):2230-7. Epub 2003/05/08. doi: 10.1001/jama.289.17.2230. PubMed PMID: 12734134.
33. Park J, Morley TS, Kim M, Clegg DJ, Scherer PE. Obesity and cancer--mechanisms underlying tumour progression and recurrence. Nat Rev Endocrinol. 2014;10(8):455-65. Epub 2014/06/18. doi: 10.1038/nrendo.2014.94. PubMed PMID: 24935119; PubMed Central PMCID: PMC4374431.
34. Statistics on Cancer Incidence 2020: Socialstyrelsen, The National Board of Health and Welfare; 2021 [updated 15 Dec 202116 Jan 2022]. Available from: https://www.socialstyrelsen.se/globalassets/sharepoint-dokument/artikelkatalog/statistik/2021-12-7701.pdf.
35. Kheirandish-Gozal L, Gozal D. Obstructive Sleep Apnea and Inflammation: Proof of Concept Based on Two Illustrative Cytokines. Int J Mol Sci. 2019;20(3). Epub 20190122. doi: 10.3390/ijms20030459. PubMed PMID: 30678164; PubMed Central PMCID: PMC6387387.
36. Unnikrishnan D, Jun J, Polotsky V. Inflammation in sleep apnea: an update. Rev Endocr Metab Disord. 2015;16(1):25-34. doi: 10.1007/s11154-014-9304-x. PubMed PMID: 25502450; PubMed Central PMCID: PMC4346503.
37. Multhoff G, Molls M, Radons J. Chronic inflammation in cancer development. Front Immunol. 2011;2:98. Epub 20120112. doi: 10.3389/fimmu.2011.00098. PubMed PMID: 22566887; PubMed Central PMCID: PMC3342348.
38. Ekstrom M, Albrecht D, Andersson S, Grote L, Karrsten Rundstrom B, Palm A, et al. Validation of the Swedevox registry of continuous positive airway pressure, long-term mechanical ventilator and long-term oxygen therapy. ERJ Open Res. 2021;7(1). Epub 2021/02/04. doi: 10.1183/23120541.00340-2020. PubMed PMID: 33532458; PubMed Central PMCID: PMC7836467 D. Albrecht has nothing to disclose. Conflict of interest: S. Andersson has nothing to disclose. Conflict of interest: L. Grote reports no conflict of interest for the actual study. Outside the submitted work, L. Grote reports a research collaboration with Bayer AG, nonfinancial support and other from Itamar Medical, Resmed, Philips and Astra Zeneca. In addition, Dr Grote has a patent on sleep apnoea therapy issued and licensed. Conflict of interest: B. Karrsten-Rundstrom has nothing to disclose. Conflict of interest: A. Palm has nothing to disclose. Conflict of interest: J. Theorell-Haglow has nothing to disclose. Conflict of interest: J. Wahlberg has nothing to disclose. Conflict of interest: B. Midgren has nothing to disclose.
39. Grote Lea. National guidlines for for treatment of sleep apnea in adults 2021 [30 april 2022]. Available from: https://sesar.registercentrum.se/riktlinjer/nationellt-vardprogram-foer-behandling-av-soemnapne-hos-vuxna/p/HkusG6OcK.
40. Tan BKJ, Teo YH, Tan NKW, Yap DWT, Sundar R, Lee CH, et al. Association of obstructive sleep apnea and nocturnal hypoxemia with all-cancer incidence and mortality: a systematic review and meta-analysis. Journal of clinical sleep medicine : JCSM : official publication of

For peer review only - http://bmjopen.bmj.com/site/about/guidelines.xhtml

the American Academy of Sleep Medicine. 2022;18(5):1427-40. doi: 10.5664/jcsm.9772. PubMed PMID: 34755597; PubMed Central PMCID: PMC9059590.
41. Axelsson Fisk S, Lindström M, Perez-Vicente R, Merlo J. Understanding the complexity of socioeconomic disparities in smoking prevalence in Sweden: a cross-sectional study applying intersectionality theory. BMJ Open. 2021;11(2):e042323. Epub 20210211. doi: 10.1136/bmjopen-2020-042323. PubMed PMID: 33574148; PubMed Central PMCID: PMC7880088.

FIGURE LEGENDS

Figure 1. Study flow chart

Figure 2. Distribution of OSA patients with (red bars) and without (blue bars) cancer diagnosis in the four ODI quartiles (x-axis)
Propensity-score matched analysis in 2,093 cases and 2,093 controls, ODI distribution differ significantly between cases and controls, p<0.001.

For peer review only

For peer review only - http://bmjopen.bmj.com/site/about/guidelines.xhtml

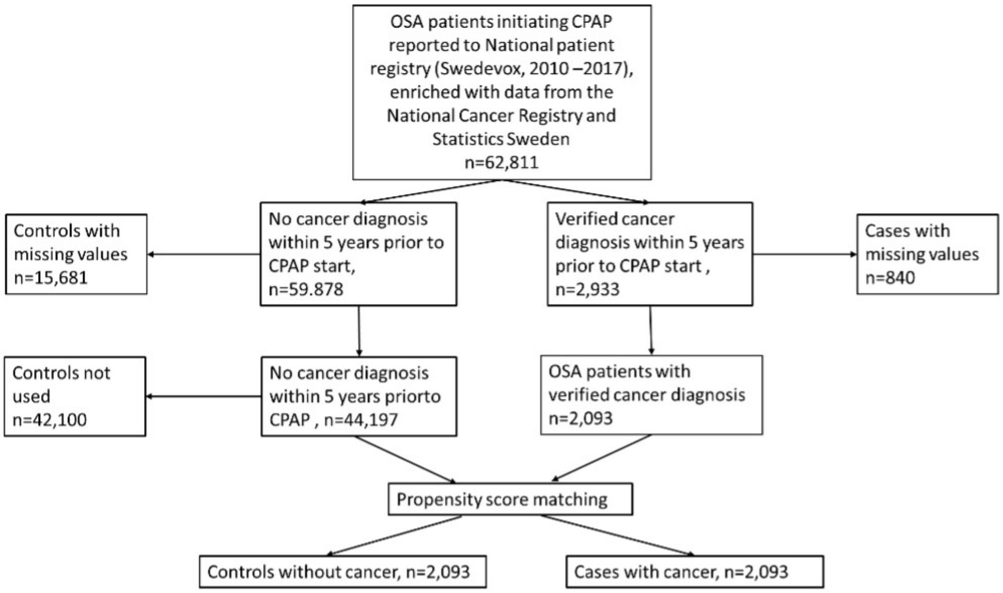

Figure 1. Study Flow Chart

167x99mm (150 x 150 DPI)

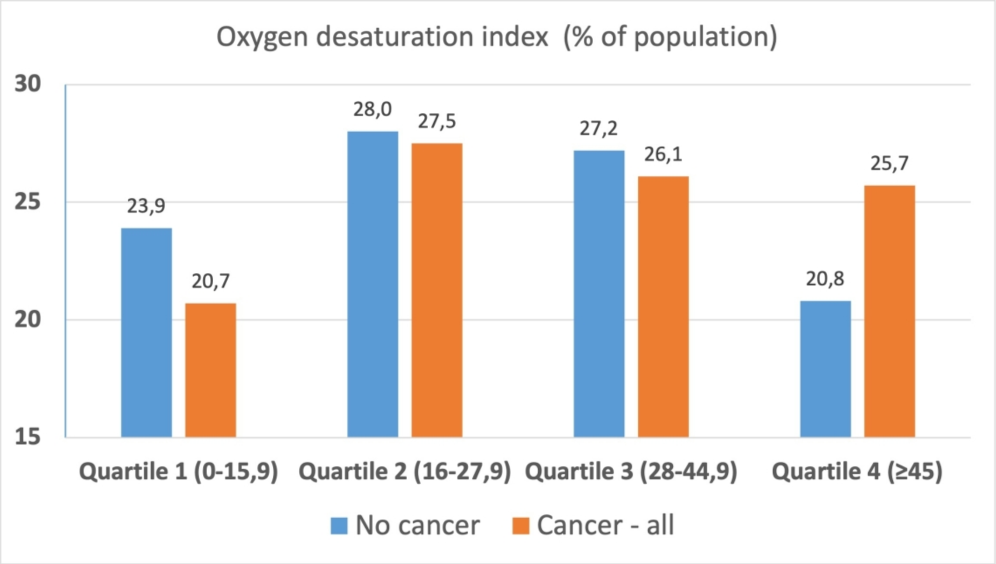

Figure 2. Distribution of OSA patients with (red bars) and without (blue bars) cancer diagnosis in the four ODI quartiles (x-axis). Propensity-score matched analysis in 2,093 cases and 2,093 controls, ODI distribution differ significantly between cases and controls, p<0.001.

160x91mm (330 x 330 DPI)

Reporting checklist for cross sectional study.

Based on the STROBE cross sectional guidelines.

Instructions to authors

Complete this checklist by entering the page numbers from your manuscript where readers will find each of the items listed below.

Your article may not currently address all the items on the checklist. Please modify your text to include the missing information. If you are certain that an item does not apply, please write "n/a" and provide a short explanation.

Upload your completed checklist as an extra file when you submit to a journal.

In your methods section, say that you used the STROBE cross sectionalreporting guidelines, and cite them as:

von Elm E, Altman DG, Egger M, Pocock SJ, Gotzsche PC, Vandenbroucke JP. The Strengthening the Reporting of Observational Studies in Epidemiology (STROBE) Statement: guidelines for reporting observational studies.

For peer review only

		Reporting Item	Page Number
**Title and abstract**			
Title	#1a	Indicate the study's design with a commonly used term in the title or the abstract	2,3
Abstract	#1b	Provide in the abstract an informative and balanced summary of what was done and what was found	3
**Introduction**			
Background / rationale	#2	Explain the scientific background and rationale for the investigation being reported	4
Objectives	#3	State specific objectives, including any prespecified hypotheses	4
**Methods**			
Study design	#4	Present key elements of study design early in the paper	5
Setting	#5	Describe the setting, locations, and relevant dates, including periods of	5-6

		recruitment, exposure, follow-up, and data collection	
Eligibility criteria	#6a	Give the eligibility criteria, and the sources and methods of selection of participants.	5-6
	#7	Clearly define all outcomes, exposures, predictors, potential confounders, and effect modifiers. Give diagnostic criteria, if applicable	5-6
Data sources / measurement	#8	For each variable of interest give sources of data and details of methods of assessment (measurement). Describe comparability of assessment methods if there is more than one group. Give information separately for for exposed and unexposed groups if applicable.	5-6
Bias	#9	Describe any efforts to address potential sources of bias	6-7
Study size	#10	Explain how the study size was arrived at	8
Quantitative variables	#11	Explain how quantitative variables were handled in the analyses. If applicable, describe which groupings were chosen, and why	6-7
Statistical methods	#12a	Describe all statistical methods, including those used to control for confounding	6-7
Statistical methods	#12b	Describe any methods used to examine subgroups and interactions	6-7
Statistical methods	#12c	Explain how missing data were addressed	6-7
Statistical methods	#12d	If applicable, describe analytical methods taking account of sampling strategy	N/A
Statistical methods	#12e	Describe any sensitivity analyses	9
**Results**			
Participants	#13a	Report numbers of individuals at each stage of study—eg numbers potentially eligible, examined for eligibility, confirmed eligible, included in the study, completing follow-up, and analysed. Give information separately for for exposed and unexposed groups if applicable.	8
Participants	#13b	Give reasons for non-participation at each stage	N/A
Participants	#13c	Consider use of a flow diagram	Figure 1

For peer review only

Descriptive data	#14a	Give characteristics of study participants (eg demographic, clinical, social) and information on exposures and potential confounders. Give information separately for exposed and unexposed groups if applicable.	8
Descriptive data	#14b	Indicate number of participants with missing data for each variable of interest	8
Outcome data	#15	Report numbers of outcome events or summary measures. Give information separately for exposed and unexposed groups if applicable.	8-9
Main results	#16a	Give unadjusted estimates and, if applicable, confounder-adjusted estimates and their precision (eg, 95% confidence interval). Make clear which confounders were adjusted for and why they were included	8-9
Main results	#16b	Report category boundaries when continuous variables were categorized	6
Main results	#16c	If relevant, consider translating estimates of relative risk into absolute risk for a meaningful time period	N/A
Other analyses	#17	Report other analyses done—e.g., analyses of subgroups and interactions, and sensitivity analyses	9
**Discussion**			
Key results	#18	Summarise key results with reference to study objectives	9
Limitations	#19	Discuss limitations of the study, taking into account sources of potential bias or imprecision. Discuss both direction and magnitude of any potential bias.	12-13
Interpretation	#20	Give a cautious overall interpretation considering objectives, limitations, multiplicity of analyses, results from similar studies, and other relevant evidence.	14
Generalisability	#21	Discuss the generalisability (external validity) of the study results	12
**Other Information**			
Funding	#22	Give the source of funding and the role of the funders for the present study and, if applicable, for the original study on which the present article is based	14-15

The STROBE checklist is distributed under the terms of the Creative Commons Attribution License CC-BY. This checklist was completed on 07. May 2022 using https://www.goodreports.org/, a tool made by the EQUATOR Network in collaboration with Penelope.ai

For peer review only
